# Mitochondria-associated programmed cell death as a therapeutic target for age-related disease

**DOI:** 10.1038/s12276-023-01046-5

**Published:** 2023-08-23

**Authors:** Thanh T. Nguyen, Shibo Wei, Thu Ha Nguyen, Yunju Jo, Yan Zhang, Wonyoung Park, Karim Gariani, Chang-Myung Oh, Hyeon Ho Kim, Ki-Tae Ha, Kyu Sang Park, Raekil Park, In-Kyu Lee, Minho Shong, Riekelt H. Houtkooper, Dongryeol Ryu

**Affiliations:** 1 Department of Biomedical Science and Engineering, Gwangju Institute of Science and Technology (GIST), Gwangju, 61005 Republic of Korea; 2 Department of Precision Medicine, Sungkyunkwan University School of Medicine, Suwon, 16419 Republic of Korea; 3 Department of Physiology, Yonsei University Wonju College of Medicine, Wonju, 26426 Republic of Korea; 4 Department of Molecular Cell Biology, Sungkyunkwan University School of Medicine, Suwon, 16419 Republic of Korea; 5 Department of Korean Medical Science, School of Korean Medicine, Pusan National University, Yangsan, 50612 Republic of Korea; 6 Service of Endocrinology, Diabetes, Nutrition and Patient Therapeutic Education, Geneva University Hospitals, Geneva, 1205 Switzerland; 7 Department of Health Sciences and Technology, Samsung Advanced Institute for Health Sciences and Technology, Sungkyunkwan University, Seoul, 06351 Republic of Korea; 8 Department of Internal Medicine, School of Medicine, Kyungpook National University, Kyungpook National University Hospital, Daegu, 41944 Republic of Korea; 9 Department of Internal Medicine, Chungnam National University School of Medicine, Daejeon, 35015 Republic of Korea; 10 Laboratory Genetic Metabolic Diseases, Amsterdam UMC Location University of Amsterdam, Meibergdreef 9, 1105 AZ Amsterdam, The Netherlands; 11 Amsterdam Gastroenterology Endocrinology and Metabolism, Amsterdam UMC Location University of Amsterdam, Meibergdreef 9, 1105 AZ Amsterdam, The Netherlands; 12 Amsterdam Cardiovascular Sciences, Amsterdam UMC Location University of Amsterdam, Meibergdreef 9, 1105 AZ Amsterdam, The Netherlands

**Keywords:** Apoptosis, Metabolic syndrome

## Abstract

Mitochondria, ubiquitous double-membrane-bound organelles, regulate energy production, support cellular activities, harbor metabolic pathways, and, paradoxically, mediate cell fate. Evidence has shown mitochondria as points of convergence for diverse cell death-inducing pathways that trigger the various mechanisms underlying apoptotic and nonapoptotic programmed cell death. Thus, dysfunctional cellular pathways eventually lead or contribute to various age-related diseases, such as neurodegenerative, cardiovascular and metabolic diseases. Thus, mitochondrion-associated programmed cell death-based treatments show great therapeutic potential, providing novel insights in clinical trials. This review discusses mitochondrial quality control networks with activity triggered by stimuli and that maintain cellular homeostasis via mitohormesis, the mitochondrial unfolded protein response, and mitophagy. The review also presents details on various forms of mitochondria-associated programmed cell death, including apoptosis, necroptosis, ferroptosis, pyroptosis, parthanatos, and paraptosis, and highlights their involvement in age-related disease pathogenesis, collectively suggesting therapeutic directions for further research.

## Age-related disease: Role of compromised control of mitochondrial quality

Therapies directed at the quality control systems of mitochondria have the potential to preserve cellular health in individuals with age-related diseases. Dongryeol Ryu from Gwangju Institute of Science and Technology in South Korea, Riekelt Houtkooper from the Amsterdam University Medical Center in The Netherlands, and colleagues review how dysfunctional mitochondria, which are responsible for producing energy in cells, contribute to cancer and many neurodegenerative, cardiovascular and metabolic conditions. When mitochondria become damaged or stressed, they can trigger cell death mechanisms via impaired quality-control networks and associated signaling pathways, further exacerbating disease processes. By gaining a deeper understanding of how mitochondrial safeguards help to maintain functional integrity, and the different ways in which mitochondria-associated cell death occurs, researchers may identify new targets for drug development.

## Introduction

**Fig. 1:**
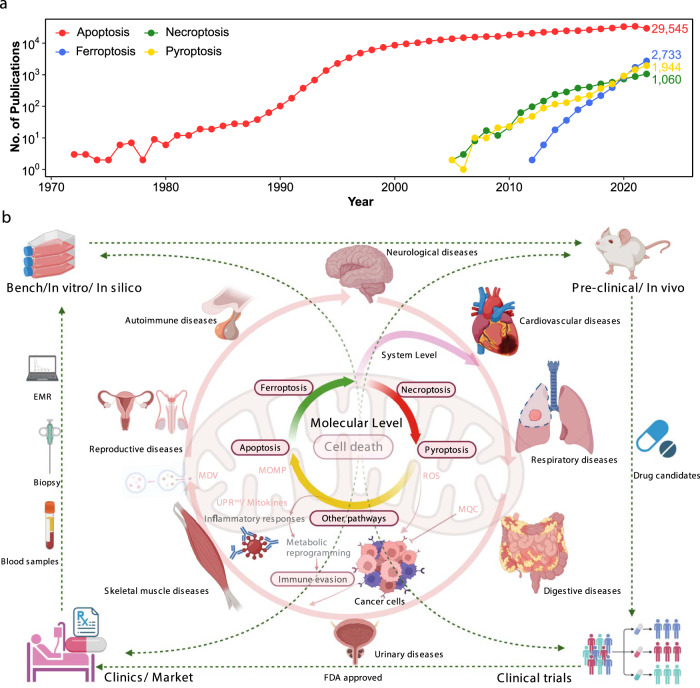
Overview of mitochondrion-associated programmed cell death and human diseases. **a** Publications of typical programmed cell death in the last 50 years. **b** Mitochondria are vital in regulating cellular metabolism and are involved in the pathogenesis and progression of numerous human diseases, directly or indirectly, through many pathways. Mitochondria are also essential in regulating cell fate via PCD, an evolutionarily conserved process in multicellular organisms that is indispensable for modulating homeostasis, containing diverse patterns, including apoptosis, necroptosis, ferroptosis, and pyroptosis. Mitochondria-associated PCD is extensively involved in the pathological progression of various disorders in different organ systems. In this regard, fresh insights into the interplay between mitochondria and PCD are expected to be inspired in disease treatment development in contexts ranging from in vitro to preclinical and clinical trials. PCD Programmed cell death, EMR Electronic medical record.

Mitochondria, at the core of cellular metabolism, play pivotal roles in key metabolic activities, from energy production to cellular signaling^1^, all of which are critical to organism health. Mitochondrial dysfunction is a profound factor contributing to aging and various age-related diseases, such as neurodegenerative, cardiovascular, and metabolic diseases. Due to their irreplaceable roles and incompletely characterized mechanisms, mitochondria have garnered increased interest in recent decades, especially in the last five years, in which more than 10,000 research papers have been published annually and in which novel protective pathways and therapeutic targets, such as mitochondrial quality control (MQC) networks^2^ and programmed cell death (PCD), have been described. As accumulated evidence indicates, in an apparent paradox, mitochondria determine cell fate by governing PCD pathways. Since its discovery as a decisive form of cell lifespan termination, PCD has garnered considerable attention. The first PCD described, apoptosis, has been the topic of research for more than 5 decades and ha been described in as many as 30,000 annual publications in a 5-year period, and surprisingly, a significant portion of these publications have characterized and substantiated the unequivocal involvement of mitochondria in apoptosis^3^. Research describing newly discovered forms of nonapoptotic PCD, such as necroptosis, ferroptosis, and pyroptosis, has been conducted for more than 20 years, and on average, more than 5,000 annual publications in this period (Fig. 1a) implicated mitochondria in non-PCD mechanisms of action^4^. However, the specific interactions between mitochondrial and PCD factors and the underlying mechanisms remain poorly characterized.

Here, we provide an overview of the current state of the field with a specific focus on regulatory mechanisms of MQC systems discovered to date and mitochondrial contributions to various forms of PCD, including the underlying molecular mechanisms and effects on human diseases. We highlight mitochondria as the primary nodes in which PCD is triggered, death signaling is propagated, cell death is realized, and the considerable impacts that cell fate exerts on the incidence of numerous diseases. We also highlight unique insights into the interactions between mitochondrial and PCD factors that have been applied for developing therapies on the basis of preclinical research in vitro and clinical trials (Fig. 1b), which may provide further direction for effective therapy.

## Mitochondrial biology

### Mitochondrial structure

**Fig. 2:**
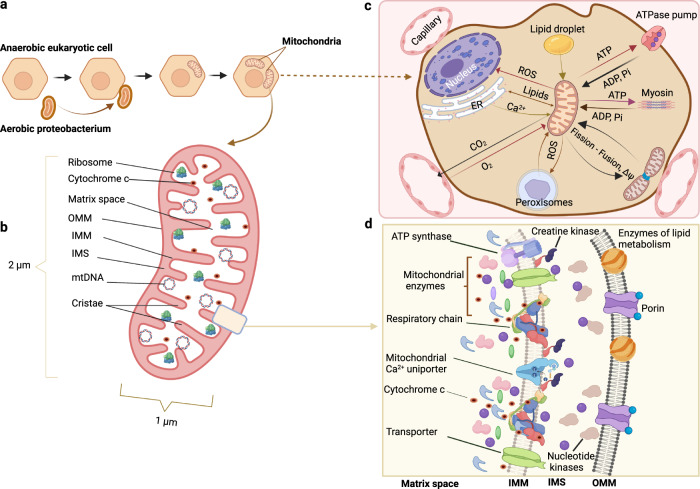
Mitochondrial structure, function, and communication with other organelles. **a**, **b** Hypothetical depiction of the origin of mitochondria from Aerobic Proteobacterium and mitochondrial simplified structure. **c** Microstructure and enzyme complexes of mitochondria. **d** Mitochondrial functions rely on cellular interactions. OMM Outer mitochondrial membrane, IMM Inner mitochondrial membrane, IMS Intermembrane mitochondrial space, mtDNA Mitochondrial DNA, ROS Reactive oxygen species, ER Endoplasmic reticulum, ATP Adenosine triphosphate.

Mitochondria, cytoplasmic organelles in most eukaryotic cells (Fig. 2a), are enclosed by two unique phospholipid membranes, the inner mitochondrial membrane (IMM) and outer mitochondrial membrane (OMM), which separate specialized and functionally compartmentalized structures, defined as the intermembrane space and matrix. These two membranes show clearly distinct chemical and molecular properties in terms of lipid composition, transmembrane protein functions, and permeability due to the organelle’s endosymbiotic origin. In contrast to the IMM, the OMM permits ions and small molecules to flow through voltage-dependent anion channels that enable only the free transport of water, gaseous substrates, and products of oxidative phosphorylation. This selectivity enables the formation of cross-membrane electrochemical gradients needed for ATP production. The IMM undergoes distinct morphological organizational changes to form densely clustered invaginations into the matrix referred to as cristae, which establish a platform for imported host-derived proteins and form crista junctions^5^ (Fig. 2b).

Mitochondria carry a unique genetic code, mitochondrial DNA (mtDNA), with 16,569 base pairs but no introns^6^. In contrast to two copies of nuclear DNA (nDNA), multiple copies of mtDNA, corresponding to the number of genes related to the energy production needed in different tissues, are present in cells. Surprisingly, during evolution, the majority of mitochondrial genes were lost or were translocated to nuclei, becoming nDNA; 37 genes, however, remained in mtDNA, and they encode 2 ribosomal RNAs and 22 transfer RNAs, which are essential components of the translational apparatus, and mtDNA encodes 13 proteins, which function as core constituents of OXPHOS complexes embedded in the IMM^7^. An estimated 1500 proteins are expressed in mitochondria, the majority of which are encoded by nDNA and translated outside of mitochondria before being transported into the organelle via specialized import pathways^8^. In mitochondria, these proteins constitute the components of the electron transport chain, metabolic pathways, etc.

### Mitochondrial function

Mitochondria are widespread intracellular organelles in virtually all eukaryotes, where they function as metabolism and signaling hubs for a broad range of cellular activities; their primary roles involve regulating energy production and cell signaling, with additional functions in mitonuclear crosstalk, such as that related to fatty acid oxidation, iron–sulfur cluster biosynthesis, and thermogenesis^9–11^ (Fig. 2c).

Because mitochondria are cell powerhouses, many catabolic pathways converge in mitochondria, particularly pathways involved in ATP production mediated through oxidative phosphorylation. Specifically, the catabolism of metabolites generates acetyl-CoA, which is oxidized in the tricarboxylic acid cycle to produce two reduced electron donors, driving the mitochondrial electron transport chain. Electrons are transferred to and flow through electron transport chain complexes, resulting in proton outflow through the IMM and formation of an electrochemical gradient; this gradient generates the proton-motive force that drives protons back to the matrix via complex V, a rotary turbine that catalyzes the synthesis of ATP^1^.

Mitochondria regulate cell signaling primarily via the influx of signaling molecules and by forming platforms on which multiple signaling interactions occur. Mitochondria modulate signaling pathways ranging from cytochrome c (cyt *c*) release to caspase activation, accompanied by signaling complex activation, immune responses, and PCD induction^4^. Mitochondria regulate the levels of intracellular molecules, including reactive oxygen species (ROS) and Ca^2+^. When Ca^2+^ accumulates in mitochondria, the activity of Ca^2+^-sensitive dehydrogenases and metabolite transporters is modulated, which accelerates oxidative metabolism (Fig. 2d). High-conductance channel-induced pore formation in the IMM, called mitochondrial permeability transition (MPT), is triggered by Ca^2+^ overload and ROS. It is linked to significant alterations in mitochondrial morphology and functional activities of various enzymes, which modulate mitochondrial respiration and ATP production^12^. ROS are produced by electron transport from redox donors to molecular oxygen, which is then converted to hydrogen peroxide and dispersed into the cytoplasm; ROS participate directly in intercellular signaling to regulate basal or adaptation responses that maintain organismal homeostasis^13^.

## Mitochondrial quality control and mitohormesis

**Fig. 3:**
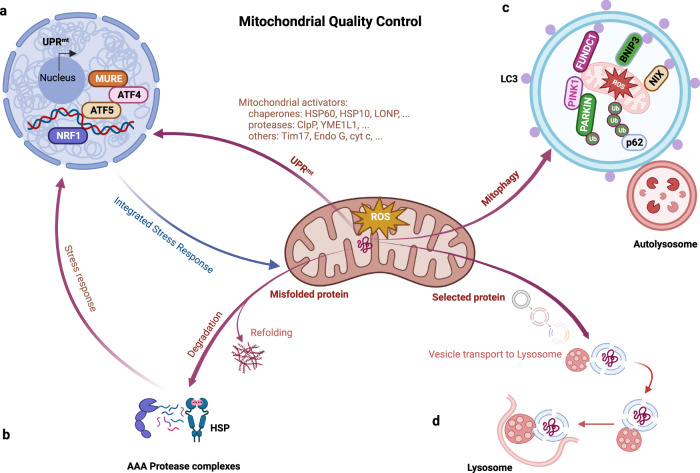
Pathways of mitochondrial quality control. **a** Activation of the UPR^mt^ pathway is triggered by the accumulation of unfolded proteins in the mitochondrial matrix, expression of mitochondrial protein import components, and upregulation of chaperones and proteases for reestablishing mitochondrial proteostasis. **b** Molecular chaperones and proteolytic enzymes in the mitochondria perform a major function in refolding or destroying mitochondrial misfolded and unfolded proteins. **c** Mitochondrial proteins are likely to be decomposed by being transported to lysosomes, where vesicles formed from mitochondrial tubules seize specific mitochondrial cargos for lysis. **d** Fragmented dysfunctional mitochondria are removed by mitophagy. Bnip3, FUNDC1, and NIX are mitophagy receptors that bind LC3 to tether mitochondria to autophagosomes. OMM proteins and Parkin are ubiquitinated when PINK1 phosphorylates proteins on the surface of depolarized mitochondria. The p62 adaptor can recognize ubiquitinated proteins to initiate mitophagy. UPR^mt^ mitochondrial Unfolded Protein Response, MURE Mitochondrial Unfolded Protein Response Element, ATF Activating Transcription Factor, NRF Nuclear Respiratory Factor, HSP Heat Shock Protein, LC3 microtubule-associated protein Light Chain 3, Bnip3 Bcl-2 interacting protein 3, Ub Ubiquitin, PINK1 PTEN-induced kinases 1.

Because mitochondria are hubs of energy production and guardian of multiple metabolic processes and cell signaling pathways, dysfunctional mitochondria cause diverse diseases across species^14,15^. To maintain functionality and ensure homeostasis, different mechanisms in cells sense and react to aberrant mitochondrial activities; these mechanisms constitute MQC systems^2^ (Fig. 3). In response to general insults, such as electron transfer chain (ETC) suppression, protein misfolding, and redox imbalances, mitochondrial integrity and metabolism are strictly regulated by DNA repair networks, antioxidants, and proteases or chaperones engaged in the mitochondrial unfolded protein response (UPR^mt^), which has been extensively described^16^. Notably, an adaptive response, mitochondria–cytosol–nucleus crosstalk, also known as mitochondrial retrograde signaling or mitohormesis, is triggered nonautonomously to attenuate cellular disorder. The coordinated interplay among crosstalk factors leads to the production of versatile mitokines, which facilitate the transcriptional upregulation of stress response proteins and promote cytoprotective and stress resistance mechanism activation^17^. However, when mitochondria are not promptly rescued from excessive damage, overexpression of certain proteotoxic signals induces the activity of additional protein quality control (PQC) networks, including the UPR^mt^ and mitophagy, through which malfunctioning mitochondria are engulfed, preserving proteostasis^18^.

### Mitokines

Mitokines, a term initially coined in the context of prolonged lifespan after mitochondrial chain disruption^16^, are currently defined as metabolic cytokines or mitochondrion-derived peptides (MDPs) that are released by cells in response to mitochondrial stresses and that affect systematic metabolism and longevity^17^. Mitokines are nucleus-encoded proteins and respond to mitochondrial insults, such as growth differentiation factor 15 (GDF15) and fibroblast growth factor 21 (FGF21), and alterations in both of these factors are involved in multiple mitochondrion-related disorders and inherited metabolic diseases^19^. Moreover, various MDPs generated by short open-reading frames (ORFs) located in the regions encoding 12 S and 16 S rRNA in mtDNA have been discovered; these mitochondrial ORFs encode 12 S rRNA-c (MOTS-c), humanin (HN), and six small HN-like peptides (SHLPs)^20^, which exhibit systemic effects such as neuroprotection, enhanced metabolism, antiapoptotic functions and increased viability. However, the specific functions underlying their activity are unclear.

#### GDF15

Several studies have indicated that GDF15, a vital humoral factor secreted from skeletal muscle and adipose tissue cells after mitochondrial stress- and UPR^mt^-induced activation, may drive mitohormesis^17^. Circulating GDF15 levels are elevated in individuals with mitochondrial diseases and subjects characterized by mitochondrial impairment or mitoribosomal protein deficiency^21^, and GDF15 systematically increases metabolism in dozens of diseases^22,23^. When overexpressed, GDF15 regulates systemic energy homeostasis, attenuates mitochondrial malfunction, and promotes metabolic reorganization and adaptability^24^. However, some reports have indicated that GDF15 exerts certain detrimental effects under pathophysiological conditions and is partially involved in muscle wasting and cachexia^25^. However, the systemic mechanisms underlying GDF15 action in mitohormesis are unclear, and further studies are needed.

#### FGF21

Research indicates that FGF21 functions as a potent mitokine, in addition to an endocrine regulator and longevity hormone, as originally described. It is secreted under mitochondrial stress conditions induced by abnormalities in the ETC or via autophagy. An in-depth study has revealed that FGF21 can also be activated in reaction to various mitochondrial stresses, including mitofusin deletion, mtDNA mutations, and mitochondrial ROS overexpression, encouraging a health-promoting response. A recent study discovered an intriguing feature FGF21: It is induced under mild-to-moderate mitochondrial stress conditions but is dispensable or overridden via whole-body metabolic adaptation under severe disease conditions^26^. However, despite these findings, which revealed a more nuanced function of FGF21, the health-promoting mitohormetic effects of FGF21 are generally recognized. Similar to GDF15, FGF21 exhibits various beneficial regulatory metabolic functions, conferring protection against hormone resistance and increasing glucolipid metabolism and lipothermogenesis, as thoroughly described in a prior study^27^. The functions of FGF21 have been confirmed by mechanistic evidence showing that FGF21 suppresses oxidative phosphorylation gene expression and rescues the ETC to activate ATF4, a transcription factor targeting the FGF21 promoter^28^ and a master regulator of the integrated stress response^29^, leading to cellular homeostasis restoration.

#### MDP

Typical MDP functions include predominantly the regulation of metabolic signaling and restoration of mitochondrial homeostasis^30^. HN was the first MDP proposed to exert cytoprotective effects on different aging diseases, such as cardiovascular diseases (CVDs), neurodegenerative diseases, and hepatic steatosis, owing to its anti-inflammatory and antiapoptotic effects that are triggered via increased Ca^2+^ flux and activation of extracellular signal-regulated kinase and downstream signal cascades^31^. MOTS-c may be transported to the nucleus and drive responsive gene expression under stress conditions via interconnections between chromatin and stress-induced transcriptional regulators^32^. MOTS-c, similar to FGF21, demonstrates dominant metabolome-protective features in multiple types of tissues with metabolic disorders, principally functioning to maintain glucolipid metabolism and regulate lipothermogenesis^33^. SHLPs, especially SHLP2 and SHLP3, play promising roles in inhibiting apoptosis by inhibiting caspase activation and ROS production and promoting adipocyte differentiation^34^.

### The UPR^mt^

The UPR^mt^ is an adaptable transcriptional process that maintains mitochondrial homeostasis. It is activated by proteotoxic stress, such as that induced by misfolded mitochondrial precursor protein deposition and protein mistargeting^35^, to limit cytosolic protein production and degrade mislocated proteins in mitochondria that cannot be transported. Both the ubiquitin‒proteasome system and mitophagy are advantageous for mitochondrial proteostasis and are mediated via the PQC system; these processes are mediated by chaperones and proteases that manage protein folding, assembly, and degradation.

The UPR^mt^ signaling pathway has been most extensively characterized in invertebrates^36^, in which signaling cascades have, however, proven to be significant different from those in mammals. The UPR^mt^ response has been preserved throughout evolution, as indicated by the levels of specific genetic markers in genetically engineered mouse models and humans. According to recent research and bioinformatics analysis, in mammals, the UPR^mt^ pathway is epigenetically regulated by histone H3 lysine 27 demethylases and characterized by the stimulation of multiple chaperones (CHOP, TRAP1, mtHSP70, and the HSP60-MSP10 multimer) and proteases (ClpP, YME1L1, and LONP1)^37^. However, the underlying mechanisms of most retrograde messengers or promotors remain poorly understood, with some studies reporting paradoxical results.

Mitochondrial stress promotes the activation of the UPR^mt^ either directly via mtDNA disorder-induced imbalance among OXPHOS complexes and indirectly via ATF4 and ATF5^38,39^ to maintain cell proliferation in an elF2α-dependent manner and as proposed mediators in cardioprotection, respectively^40^.

### Mitophagy

Mitophagy is an evolutionarily conserved mechanism in which identification and clearance of mitochondria targeted for elimination in response to misfolded mitochondrial protein accumulation or mitochondrial depolarization are realized, and it is required for MQC maintenance^2^. In this process, distinct mitophagy pathways are likely to be involved in diverse mechanisms and coordinate to initiate rapid responses to overload-induced damage with highly specialized functions to preserve mitochondrial homeostasis^41^.

#### Parkin and PINK

A pair of well-characterized mitophagy mediators involved in one mechanism is PTEN-induced putative kinase 1 (PINK1) and E3 ubiquitin ligase Parkin^41^. Recent evidence highlights PINK1 as a sensor or mitochondrial damage and in a surveillance mechanism that maintains mitochondrial fitness; it recruits Parkin to damaged mitochondria, promoting autophagic clearance in a Parkin-dependent ubiquitination process^42^. This pathway is also involved in regulating mitochondrial dynamics through fission and fusion. Consistent with these functions, Parkin is translocated into mitochondria after mitochondrial membrane depolarization and induces mitofusin polyubiquitination, targeting it for proteasomal degradation to prevent defective mitochondria from fusing with functioning mitochondrial networks; mitofusin polyubiquitination is required for subsequent degradation by mitophagy^43^.

#### BNIP3 and BNIP3L

Bnip3, in the B-cell lymphoma 2 (Bcl-2) family of PCD-regulating factors, plays a paradoxical but pivotal role in regulating diverse mitochondrial functions, such as oxidation, calcium overload, ATP shortage, and secondary mitochondrial disorders^44^. Similar to the conventional hypoxia-responsive regulator, Bnip3 induces mitophagy after autophagy stimulation and is involved in mitochondrial fragmentation, triggering mitochondrial depolarization, the subsequent sequestration of disordered mitochondria into autophagosomes, and ultimately, mitochondrial elimination.

Another mitophagy receptor, Bnip3-like (Bnip3L) or NIX, has recently been shown to exhibit functions in addition to its function as a BH3-only proapoptotic factor^45^. Bnip3L promotes mitophagy by facilitating autophagosome aggregation via disruption of the Bcl-2-BECN1 complex and, more directly, by recruiting autophagosomes to mitochondria via interplay with proteins in the Atg8 family.

#### FUNDC1

FUN14 domain-containing 1 (FUNDC1) was originally identified as a hypoxia-induced mitophagy modulator that interacts with and recruits LC3 to mitochondria to induce mitophagy mediated by various upstream phosphorylases or phosphatases that regulate mitophagy^46^. In-depth studies have demonstrated that FUNDC1 mediates mitophagy and mitochondrial fission or fusion, regulates the coupling of the double mitochondrial membranes to promote MQC, and regulates mitochondrial dynamics by interacting with both DNM1L/dynamin-related protein 1 (DRP1) and optic atrophy 1 (OPA1) in response to stresses such those associated with energy metabolism or oxidation^47^.

## Mitochondria-associated programmed cell death

PCD is an essential feature of multicellular organism development and a major cause of degenerative diseases. As the field expands, pathways that coordinate diverse cell death pathways to preserve cellular homeostasis are being discovered^48^. PCD manifests as alterations in macroscopic morphotypes. In addition to manifestations of dead cells and their fragments before elimination, morphological characteristics of dying cells have typically been used to identify cell death modalities.

### Apoptosis

Apoptosis is an outcome of a rapid response to stimuli, and it is morphologically characterized by cell shrinkage, nuclear pyknosis, and karyorrhexis, culminating in the formation of apoptotic bodies that are eventually engulfed by resident phagocytic cells^49^. Apoptosis is critical for physiological homeostasis in almost every organ system and is induced by multiple stimuli, such as hypoxic, immune reaction, ischemic, and infectious factors.

The central signaling pathway of apoptosis includes a set of cysteine proteases called cysteinyl aspartate proteinases (caspases), which are activated by proteolysis and processing cascades triggered by proapoptotic signaling^50^. To date, approximately 14 mammalian caspases have been discovered and classified into three categories: apoptotic effectors (caspase-3, -6, and -7), apoptotic initiators (caspase-2, -8, -9, and -10), and inflammatory caspases^50^. The former two sets of caspases interact and determine apoptosis, which is mediated by the cleavage of certain substrates by effector caspase-3 and -7 when initiator caspase-8, -9, and -10 are activated in response to cues from upstream adaptor molecules. In contrast, instead of functioning in apoptosis, inflammatory caspases are involved in inflammatory cytokine signaling and other forms of PCD, such as pyroptosis.

Cyt *c* is a heme protein synthesized as an apoprotein in the cytoplasm. It is then translocated to mitochondrial intermembranous and intercristal regions, where it functions as an important hub in the respiratory chain^51^. Upon activation, Cyt *c* is released into the cytoplasm, where it facilitates the allosteric activation and oligomerization of the adaptor molecule apoptosis-protease-activating factor 1 and induces heptameric structure assembly, called an apoptosome. This site promotes caspase-9 mutation to induce subsequent caspase-activation cascades, and then, resident phagocytes degrade apoptotic cell remnants^50^. A conformational change in the protein caused by the connection of Cyt *c* with cardiolipin (Cl)-specific phospholipids in the mitochondrion enables it to function as a peroxidase to induce Cl oxidation, which triggers a cascade of events that results in apoptosis.

The Bcl-2 family, a set of 25 cytoplasmic proteins, has been identified as another driver of apoptosis. Based on their distinct modes of action, Bcl-2 proteins are classified into 3 groups, proapoptotic three-domain proteins (Bax and Bak), proapoptotic BH3-only proteins (tBid, Bad, NOXA, PUMA, and Bim), and antiapoptotic four-domain proteins (Bcl-2 and Bcl-xL). The anti-apoptotic family members mainly antagonize the effects of proapoptotic members by preventing homo- and heterodimeric interactions to maintain mitochondrial membrane integrity^51,52^. Normally, Bak is translocated to mitochondria and inactivates Bax in the cytoplasm. However, following activation, Bax is inserted into the OMM, accompanied by Bak, and they are directly bound and activated by BH3-only proteins in the hydrophobic groove, leading to extensive conformational alteration of the OMM^50^.

**Fig. 4:**
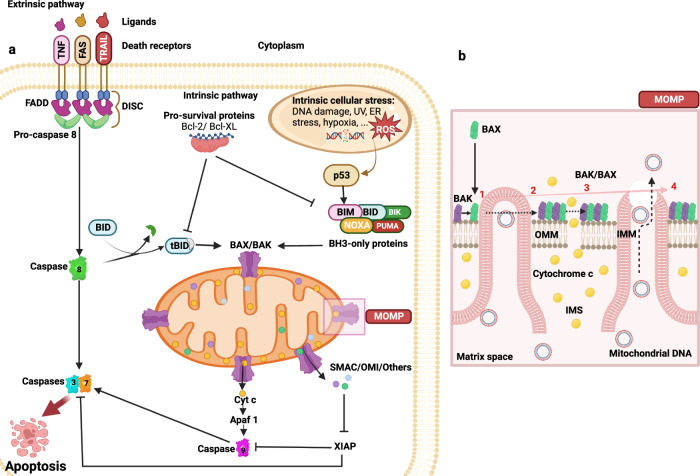
Apoptotic signaling pathways. **a** In the extrinsic pathways, death receptor ligands bind to members of the death receptor family to function, for instance, TNF, FAS, and TRAIL receptors. Numerous diverse stressors, such as DNA damage, the removal of growth factors, and mitotic arrest, activate the intrinsic apoptotic pathway, which in turn activates BH3-only members. By activating the effector proapoptotic Bcl-2 proteins Bax and Bak and inhibiting antiapoptotic Bcl-2 proteins, BH3-only proteins cause MOMP. In particular, cyt *c* is activated by releasing proteins from the mitochondrial intermembrane gap. The heptameric structure known as the apoptosome is created when cyt *c* interacts with APAF1. As a result, caspase-9 is recruited and activated, which cleaves and activates caspase-3 and -7. The caspase inhibitor XIAP is blocked by proteins such as SMAC, which are released due to MOMP, promoting apoptosis. The extrinsic and intrinsic apoptotic pathways are linked via caspase-8 cleavage and activation of the BH3-only protein Bid. **b** In normal cases, Bax localizes to the cytoplasm and Bak to the mitochondria. By interacting with BH3-only proteins, Bax and Bak can be directly activated during apoptosis, which leads to their stabilization on the OMM. Their dimer further oligomerizes into a higher-order multimer, contributing to the release of cyt *c* and other IMS proteins. Over time, Bax and Bak accumulate with macropore formation, which allows the IMM to protrude through the OMM, after which the IMM herniates and ruptures, eventually releasing mtDNA. TNF Tumor Necrosis Factor, FADD Fas-Associated Protein with DD, DISC Death-Inducing Signaling Complex, TRAIL Tumor necrosis factor-Related Apoptosis-Inducing Ligand, SMAC Second Mitochondria-derived Activator of Caspases, tBid truncated Bid, Apaf1 Apoptotic protease-activating Factor 1, XIAP X-linked Inhibitor of Apoptosis Protein, PUMA p53 Upregulated Modulator of Apoptosis, UV Ultraviolet.

Apoptosis is a genetically specified program mediated by a plethora of molecular pathways. Two of these pathways, the extrinsic and intrinsic pathways, have been extensively described as distinct but ultimately convergent processes that culminate in caspase activation^53^. The extrinsic pathway, or death receptor pathway, drives apoptosis when extracellular ligands bind cognate transmembrane death receptors, including Fas, TNF, or TRAIL (Fig. 4a). Death ligand stimulation causes the receptors to oligomerize and recruit caspase-8 and the Fas-associated death domain, promoting a the formation of death-inducing complex that, in turn, triggers the downstream caspase cascade leading to apoptotic PCD^54^. In contrast, activation of caspase-8 can drive Bid activity in the intrinsic pathway, also termed the mitochondrial or Bcl-2-regulated pathway, as bid is required for apoptotic signal amplification^52^. Intrinsic apoptosis is triggered by irreversible mitochondrial outer membrane permeabilization (MOMP), which is caused either through pore formation mediated by the Bcl-2 family, especially proapoptotic Bak and Bax, or after the MPT following the permeability transition pore (mPTP) opening^50,52^ (Fig. 4a).

An increasing body of evidence suggests that OMM integrity is the determining factor that determines cell survival or death. MOMP is the cause of primed cell apoptosis^55^ and is classified into caspase-dependent and caspase-independent forms. Generally, antiapoptotic proteins in the Bcl-2 family reside mainly on the OMM, where they protect mitochondria from factors that induce permeability, likely by coupling to and neutralizing proapoptotic factors. However, external stimuli, such as growth factor withdrawal and diverse cytotoxic insults, or internal stimuli, such as ROS, DNA damage, and hypoxia, activate proapoptotic BH3-only proteins, which exert their effects via two disparate mechanisms. Some of these proteins, such as Bad, preferentially engage with antiapoptotic proteins to release them from proapoptotic proteins to which they are bound, a process that is indispensable for morphogenesis because it induces MOMP. Other proteins, such as tBid, directly initiate proapoptotic proteins to induce MOMP by promoting Bax transport to the OMM or exert localized effects on Bak^56^. Bax and Bak collaboratively induce pore formation in the OMM, and the size of these pores vary depending on the Bax concentration and degree to which Bax assembles into rings and arcs in apoptotic mitochondria, where they support permeabilization via lipidic pore formation. As explained above, cyt *c* is released from mitochondria following MOMP and triggers caspase responses through apoptosome assembly, culminating in cellular disintegration; this series of events constitutes the caspase-dependent pathway^57^. Notably, the position of cyt *c* within the IMM and cristae does not promote its rapid release via MOMP (Fig. 4b). To enable cyt *c* transport out of mitochondria, cristae are reconfigured in a proapoptotic Bcl-2-dependent manner; specifically, Bim and Bak widen the pores to facilitate the escape of the bulk of mitochondrial cyt *c* from the intermembrane mitochondrial space (IMS), ensuring caspase activation^4^.

The activation of another caspase-dependent pathway is mediated by mPTP, which is formed by the fusion of the outer and inner leaflets of protein-stabilized membranes. mPTP is a multimeric complex harboring a voltage-dependent anion channel (VDAC) that resides in the OMM, adenine nucleotide translocase, an IMM integral protein, and the matrix protein cyclophilin D (CyPD). Among these proteins, VDAC is a mitochondrial porin that mediates Ca^2+^ movement into the IMS, and impaired Ca^2+^ transport regulation leads to matrix Ca^2+^ overload and activation of highly conductive mPTP^58^. This process is enhanced by CyPD, which increases mitochondria sensitivity to Ca^2+^, or by direct regulation of MPT^55^. mPTP opening can be enhanced by other factors, including ROS, inorganic phosphate, low pH, and ATP depletion, as well as factors including the membrane lipid environment and interorganelle communications. The deletion of Bax and Bak inhibits mitochondrial swelling and rupture after mPTP opening, indicating that some of the proteins implicated in mitochondrion-associated apoptosis may also affect the mPTP^4^.

Although numerous studies have been devoted to investigating the caspase-dependent apoptosis pathway, in each case, the overall picture remains hazy but the outline is gradually appearing. The latest research indicates that, in addition to Bcl-2 family proteins, the Bcl-2-related ovarian killer (BOK) functions as a non-Bcl-2 effector of apoptosis and is considered to play roles that overlap with that of Bcl-2 in MOMP induction, even when Bax and Bak are lacking^52^. BOK, a highly conserved protein with high similarity to Bax and Bak, is ubiquitously expressed. BOK has been shown to exert a proapoptotic effect on mitochondrial fission and morphology and thus affects MOMP. After cyt *c* release, another component, the second mitochondrion-derived activator of caspase, is also released from mitochondria into the cytoplasm, where it binds to apoptosis-protein inhibitors and leads to caspase activation^50^.

Although caspases support MOMP induced wave propagation, the inhibition of caspase activation after exposure to mitochondrial apoptosis stimulation exerted no impact on the kinetics or extent of MOMP in cells; this is an alternate mechanism underlying MOMP called the caspase-independent pathway. Underlying the maladaptive response to the irreversible loss of mitochondrial function, some caspase-independent death effectors, including endonuclease G and apoptosis-inducing factor (AIF), are translocated to the cytoplasm and, ultimately, the nucleus, where they contribute to DNA fragmentation and chromatin condensation^59^.

One thought-provoking caveat regarding cyt *c* release suggests that most cyt *c* is segregated within mitochondrial cristae. During MOMP, despite the pores opening on the OMM, the mechanism by which cyt *c* escapes from the IMS is unclear. Consistent with cyt *c* escape, cyt *c* mobilization is controlled following MOMP, and caspase activation is changed, implying an additional secondary mechanism, which is thought to be associated with cristae remodeling, modulating this process^60^. Mechanically, several BH3-only proteins, including Bid, Bik, and Bim, have been proven to initiate cristae remodeling in a MOMP-independent manner through Bax/Bak-independent or Bax/Bak-dependent pathways; this remodeling requires that the mitochondrial fission protein DRP1 level, as well as the GTPase OPA1 levels to some extent, be regulated^4,52^. OPA1 regulates IMM fusion and the size of crista junctions, while OPA1 oligomers restrict the size of the junctions, whereas OPA1 oligomer disintegration promotes junction widening. As auxiliary factors, ubiquitin-like proteins modify DRP1, stabilizing the binding sites between mitochondria and the endoplasmic reticulum (ER) membranes, where Ca^2+^ is pumped back into mitochondria to promote cristae remodeling^61^.

### Nonapoptotic PCD

Recent research on apoptosis has focused on the homeostasis and development of eukaryotic organisms. However, because mitochondria are sites of diverse cell death-promoting signaling convergence, various nonapoptotic PCD forms have been discovered. These modalities, which differ from apoptosis in their specific morphological or mechanical characteristics, are either triggered independently of apoptosis or, more frequently, kill cells as a backup mechanism when apoptosis is inhibited^62^.

#### Necroptosis

Inhibiting apoptosis cannot entirely stop PCD, but it leads to a transitions to a caspase-independent process with morphological traits comparable to those of necrosis called necroptosis^3^. Apoptosis and necroptosis differ in several respects. Morphologically, the cell membranes of cells undergoing apoptosis are preserved. In contrast, the plasma membranes of necroptotic cells are disrupted, which is accompanied by organelle swelling, nuclear condensation, chromatin digestion, DNA hydrolysis, and eventually cell lysis.

**Fig. 5:**
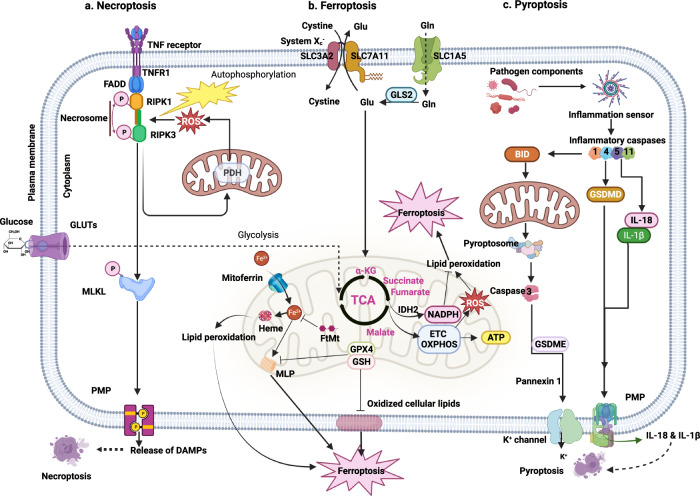
Mitochondria-associated nonapoptotic cell death. **a** Necroptosis is formed from necroptosis-driving factors such as TNF α binding with complementary receptors to recruit RIPK1, where the engagement renders RIPK1 and RIPK3 activated, which fuels necrosome configuration. The necrosome thereafter phosphorylates and activates MLKL, which is translocated to and permeabilizes the plasma membrane and permits DAMP release. **b** Ferroptosis is driven by iron-dependent lipid peroxidation and ROS overexpression. GPX4 converts excessive peroxides into lipid alcohols to mitigate this process under normal conditions; however, once stimuli occur, overexpressed superoxide from OXPHOS complexes reacts with ferrous ions to yield a slew of unstable radicals, leading to excessive ROS and, afterward, lipid peroxidation reboots to induce ferroptosis. Another route to ferroptosis is the buildup of lipid peroxide by circumstances such as cysteine deprivation, which induces the breakdown of certain iron-binding proteins, including FtMt and heme, to free iron and ROS, therefore triggering nearby MLP and hence ferroptosis. **c** Pyroptosis is initiated by the pathogen component-activated inflammatory caspase response, which not only converts IL‐1β and IL‐18 to mature forms but also cleaves GSDMD to facilitate pore penetration and enable IL-1β and IL-18 release, thus resulting in cell swelling and pyroptosis. Apart from these, inflammatory stimuli also lead to pyroptosome-mediated caspase-3 activation and GSDME cleavage to unleash membrane permeabilization to favor the release of ions and mtDNA to induce pyroptosis. DAMPs Damage-Associated Molecular Patterns, MLKL Mixed-Lineage Kinase Domain-Like pseudokinase, PDH pyruvate dehydrogenase, GPX4 Glutathione Peroxidase 4, TCA Tricarboxylic acid cycle, ETC Electron Transport Chain, GSH Glutathione, MLP Membrane Lipid Peroxidation, FtMt Mitochondrial Ferritin, α-KG Alpha-ketoglutarate, IDH2 Isocitrate Dehydrogenase 2, NADH Nicotinamide Adenine Dinucleotide Dehydrogenase, OXPHOS mitochondrial Oxidative Phosphorylation System, GSDMD Gasdermin D, GSDME Gasdermin E, IL-18 Interleukin-18, IL-1β Interleukin-1β.

Studies have revealed certain mechanisms by which tumor necrosis factor (TNFα) plays a critical role in driving necroptosis. When caspase-8 is inhibited, TNFα binds with a complementary receptor, forming a transitory membrane signaling complex containing TRADD, an adapter molecule that mediated the translocation of receptor-interacting protein kinase (RIPK) 1 to TNF, which activates RIPK1 and RIPK3, which assemble into a necrosome complex^63^. The necrosome then phosphorylates and stimulates a mixed-lineage kinase domain-like pseudokinase, which is transported to the plasma membrane and induces permeability to kill cells^64^. Recent research has linked necroptosis to mitochondria, with convincing evidence showing that progressive mitochondrial dysfunction during aging may predispose cells to undergo necroptosis. Mitochondrial ROS promote the onset of necroptosis by increasing RIPK1 autophosphorylation, resulting in RIPK3 recruitment and necrosome assembly^65^. Similarly, RIPK3 promotes mitochondrial energy metabolism and activates mitochondrial ROS generation, increasing the necrosome assembly rate and RIPK3 activity^66^ (Fig. 5a). In addition, RIPK3 can induce mitochondrial damage by upregulating the expression of mitochondrial NADPH oxidase 4 through posttranscriptional modification; other underlying mechanisms have yet to be further explored.

#### Ferroptosis

Ferroptosis is the novel term coined to explain an oxidation-related PCD modality driven by iron-dependent lipid peroxidation that has been proven to promote healthspan when inhibited^67^. Since mitochondria, the major organelles involved in iron utilization and catalytic and harboring anabolic pathways, play pivotal roles in iron homeostasis as well as substance and energy metabolism, speculation that mitochondria are regulatory hubs of ferroptosis is reasonable. This supposition is supported by the morphological features of ferroptosis, which include increased mitochondrial membrane condensation and density, reduced overall volume and number of mitochondria cristae, and loss of OMM integrity^68^.

ROS generation is one of the mechanisms governing ferroptosis. Under normal circumstances, cells harbor intrinsic defensive systems for detoxifying lipid peroxides, predominantly via glutathione peroxidase 4 (GPX4), which converts excess peroxides into lipid alcohols to mitigate ferroptosis. However, under conditions of mitochondrial stress or pathology, excess superoxide produced by OXPHOS complexes is converted to hydrogen peroxide, which then reacts with ferrous ions to yield many unstable radicals. Once the antioxidation ability of GPX4 is surpassed, excessive ROS are likely to disrupt cellular redox homeostasis and induce ferroptosis^69^. Another ferroptosis pathway involves lipid peroxide accumulation after cysteine deprivation, which increases the glutaminolysis rate and, as a result, accelerates mitochondrial respiration by activating the tricarboxylic acid cycle. Notably, this TCA cycle acceleration results in mitochondrial hyperpolarization and ROS overproduction^70^ (Fig. 5b).

#### Pyroptosis

Pyroptosis is an inflammatory PCD culminating in the permeabilization of the plasma membrane, causing leakage of cellular contents, including pro‐inflammatory mediators, especially IL‐1β and IL‐18, which are critical to induction of the innate immune response at infection sites, modulating adaptable immunity and amplifying cytolytic effects^71^. Principally operating as an innate immunological response to pathogens and oxidative stress, pyroptosis is induced after inflammasome formation and caspase-dependent cleavage of gasdermin D (GSDMD). This mechanism is classified into the caspase‐1-dependent and caspase‐independent pathways, with the latter involving caspase‐4 and caspase‐5 (Fig. 5c).

In a caspase-1-dependent manner, inflammasomes are formed when a class of pathogen-associated molecular patterns, such as pyrin domain-containing 3 (NLRP3), is recognized. These pathogen-associated molecular patterns are integrated with apoptosis-associated speck-like protein (ASC), which comprises a caspase activation and recruitment domain (CARD), to recruit pro-caspase-1 and thus drive inflammasome formation^72^. In quick succession, caspase-1 is activated, forming mature cleaved caspase-1. Then, activated caspase-1 not only converts precursors IL‐1β and IL-18 into mature proteins^73^ but also cleaves GSDMD to generate N-GSDMD, which initiates MOMP and induces IL-1β and IL-18 release, jointly forcing cell swelling and pyroptosis^74^. Notably, activation of caspase-1 can inhibit mitophagy and further increase mitochondrial damage^75^. Additionally, NLRP3 activation requires Ca^2+^ conduction; however, Ca^2+^ overload causes mitochondrial damage, which is followed by ROS overproduction and then activation of the NLRP3 inflammasome. In contrast, NLRP3 inhibition can rescue mitochondrial structure by inhibiting substances that induce mitochondrial damage^76^.

In contrast, caspase-4 and caspase-5 can be independently triggered after interacting with intracellular lipopolysaccharide via its CARD to regulate the maturation and secretion of IL-1β and IL-18 in some cases or to cleave GSDMD directly to induce NLRP3 inflammasome development, eventually leading to pyroptosis^77^. A recent report revealed that inflammatory stimuli also lead to mitochondrial damage and subsequent activated caspase-3-mediated gasdermin E (GSDME) cleavage, which drives MOMP in parallel with GSDMD cleavage to favor mtDNA release coupled with ROS production, inducing pyroptosis^78^.

#### Parthanatos

Parthanatos is a recently characterized PCD that is induced following the hyperactivation of DNA damage-responsive enzymes, especially poly (ADP-ribose) polymerase 1 (PARP1)^79^. The mechanisms governing this process have been partially identified. In the normal state, the activation of PARP1 forms a line of defense against the effects of mild stimuli such as high ROS and Ca^2+^ concentrations, enhancing the restoration of cellular homeostasis. However, PARP1 overactivation involves considerable consumption of cellular NAD^+^, a critical metabolite involved in a majority of cellular metabolism pathways^80,81^, inducing an energy crisis^82^. This crisis is exacerbated by the release of a PAR polymer from the nucleus that is toxic to mitochondria and the translocation of AIF to the nucleus, eventually inducing large-scale DNA fragmentation and chromatin condensation.

Parthanatos is similar to necroptosis to certain respects. Although the extent of their similarities is unclear, a cell undergoing parthanatos shows necroptosis-like morphological change, with PARP1 overexpression associated with rare apoptotic body necrosis. This change highlights the possibility that PARP1 is an active effector of RIPK initiators, presumably involved in mediating necroptosis downstream of RIPK1 and RIPK3 when activated; however, further exploration is still needed.

#### Paraptosis

Paraptosis is another recently described PCD frequently mediated by mitogen-activated protein kinases via an unknown mechanism^83^. Little is understood about the mechanisms underlying paraptosis. In contrast, several recent studies have indicated that it is characterized by cytoplasm and intracellular organelles vacuolization through the dilatation of mitochondria and the ER. Perturbations of ion homeostasis or cellular proteostasis are critical to paraptosis, possibly due to Ca^2+^ translocation from the ER to mitochondria, stimulating mitochondrial dilatation^84^. Although simple mechanisms underlying paraptosis have been outlined, they have yet to be studied at the molecular level.

## Diseases associated with mitochondrial cell death

Increasing evidence has highlighted the critical roles of mitochondrion-associated PCD in the pathogenesis of multiple organ systems (Table 1). Multifaced treatments targeting cell death mediators in clinical trials have been beneficial for multiple disorders. Although many PCD diseases are worthy of detailed discussion, herein, we focus on cancer and neurodegenerative, cardiovascular, and metabolic diseases, as they are the most prevalent diseases and the most recently and extensively studied. However, we briefly introduce the underlying associations among different PCD modalities and other typical diseases, including digestive diseases, inflammatory conditions, and infections.

**Table. 1:** Mitochondrion-associated programmed cell death in human diseases.

Type of cell death	Diseases	Mechanism	Treatment strategy
**Apoptosis** Release of proteins from the mitochondrial intermembrane space (IMS); Respiratory chain inhibition. PMID: 22683550	**Cancer** Breast cancer, Lung cancer, Kidney cancer, Ovarain and uterus cancer, Central nervous system (CNS), Gastro-enteric trait, Head and neck, Melanoma, Lymphomas, Leukemia. PMID: 21943236 PMID: 32203277	**Impaired death receptor signaling** - Reduced expression of death receptors - Reduced death signaling - Expression of decoy receptors without a death domain **Imbalance of proapoptotic and anti-apoptotic protein levels** - Overexpression of anti-apoptotic proteins - Low proapoptotic protein expression - Group I: Bcl-2, Bcl-xL, Mcl-1, Bcl-w, A1/BF-1, BclB/Bcl2L10 - Group II: Bid, Bim, Puma, Noxa, Bad, Bmf, Hrk, Bik - Group III: Bax, Bak, Bok/Mtd **Reduced caspase function** **Increased IAP expression** **Defects/mutations in p53**	**Targeting Bcl-2 family** - Agents that target the Bcl-2 family proteins - Silencing the Bcl family anti-apoptotic protein/genes **Targeting IAPS** - Targeting XIAP - Targeting Survivin **Targeting caspases** - Caspase-based drug therapy - Caspase-based gene therapy **Targeting p53** - p53-based gene therapy - p53-based drug therapy - p53-based immunotherapy
	**Neurodegenerative diseases** Alzheimer’s (AD), Parkinson’s (PD), Huntington’s (HD), Amyotrophic lateral sclerosis (ALS) Stroke. PMID: 34099897	**Activation of intrinsic apoptotic signaling pathway:** - Inhibition of anti-apoptotic Bcl-2 protein expression mediated by intrinsic apoptotic factors (DNA damage, oncogene activation, BH2-only proapoptotic proteins) - Release of cell death effectors (Bax and Bak) - Promote MOMP - Release apoptogenic factors and initiate a cascade of caspase **Activation of extrinsic apoptotic signaling pathway:** - Stimulation of death receptors (TNF, FAS, DR4, DR5) by ligand binding - Recruit adaptor protein (FADD, TRADD) - Activate caspase cascade **ALS**: oxidative stress, excitotoxicity, mitochondrial dysfunction, reduced anti-apoptotic Bcl-1 level, and direct roles played by superoxide dismutase 1, the BH3-only family protein BIM, overexpression of Bcl-2. **AD**: p53-dependent transcriptional upregulation of Bax and reduction in Bcl-2 and Bcl-xL levels: increased levels of amyloid-β protein, Tau, Apolipoprotein E. **HD**: The role of gene mutations in mitochondrial functions (PRKN, LRRK2, PINK1, and PARK7) increases BAK-mediated apoptosis.	**Caspases Inhibitors** - Inhibitors of MLK-JNK and MLK-p38 MAPK pathways - Inhibitors of cell cycle proteins - Inhibitors of p53 - Inhibitors of GAPDH - Inhibitors of GSK3 - Inhibitors of c-Raf - Inhibition or genetic ablation of RIPK1
	**Cardiovascular diseases** Ischemia, heart failure.	**Activation of both intrinsic and extrinsic signaling pathways** **Heart Failure:** - Extrinsic apoptotic pathway plays an important pathophysiological role (FADD) - TNF-α expression is upregulated in heart failure and is detrimental to the heart **Ischemic heart disease** - The intrinsic apoptosis signaling pathway plays a central role in ischemic heart disease - FAS, but not TNFR, is the primary factor activating the extrinsic apoptotic signaling pathway	**Inhibitors:** - Caspase - Protease - PARP-1 - Growth factors - Antioxidants
	**Infectious diseases** Bacterial, viral.	**Bacterial infection:** - Intrinsic apoptotic signaling pathway - NF-κB-dependent pro-survival pathway - Inflammasome-dependent cell death pathway **Viral infection:** - Apoptosis induced by viral RNA and DNA fragments (activates IRF-2 leading to anti-viral response and apoptosis) - Immune response-driven cell killing mechanism induction (extrinsic apoptotic signaling pathway - stimulate death receptors Fas and TNFR; intrinsic apoptotic signaling pathway - Granzyme-B released by CTLs and NK cells results in Bid cleavage, Bax, and Bak activation) - Anti-apoptotic proteins encoded by viruses inhibit intrinsic or extrinsic apoptotic signaling pathways; these viruses include HPV (E6, ICP10), gamma Herpes viruses (vFLIP), MCMV (M36), Cowpox virus (CrmA), HTLV-1 (TAX)	**The cGAS–STING signaling pathway drives inflammation** **Targeting Bcl-2 family members**
	**Autoimmune diseases** Systemic lupus erythematosus, Autoimmune lymphoproliferative syndrome, Rheumatoid arthritis, Thyroiditis	**Impaired apoptosis during immune responses** **Apoptosis rate is high in nonimmune tissue due to elevated levels of autoantigens**. **SLE:** - Overexpression of Bcl-2 or deficiency of Bim in lymphocytes - Decreased levels of Fas or mutations in FasL in lymphocytes - Reduced C1 and DNase I activity. **ALPS:** - Mutation or defects in Fas, FasL, or caspase-10. **MS:** - Impaired apoptotic elimination of autoreactive immune cells in the periphery and CNS - Increase IAP levels in monocytes and T cells. **Thyroiditis:** - TSH inhibits Fas expression - Antibodies to TSH receptors inhibit Fas-mediated apoptosis - Soluble Fas level is increased in untreated Graves’ disease - Interleukin 1β and TNF-α induce the production of soluble Fas - Fas, DR4, and DR5 are expressed in the thyroid carcinoma cell line - Mdm2 promotes apoptosis in human medullary thyroid carcinoma cells deficient in p53.	**Modulation of the Fas pathway** - Treatment with antigen-coupled cells - Recombinant human TRAIL
	**Metabolic diseases** Obesity, NAFLD, Diabetes (T1D, T2D)	**Obesity and NASH:** - Caspase-dependent apoptotic cell death (extrinsic and intrinsic) - Increased caspase-3, -7, and -9 protein activity levels and decreased phosphorylation of the anti-apoptotic Bcl2 protein - Extrinsic apoptosis involves ligand-dependent activation of cell death receptors (Fas, TNF-α and TRAIL) and then triggers the formation of DISC and activates caspase-8 - Caspase-8 activates caspase-3 or cleaves Bid to trigger MOMP - FFA triggers extrinsic apoptosis by stimulating TRAIL death receptor 2-mediated signaling. **T1D:** - CD8 + T cells are activated by recognition of β-cell antigen and induce via β-cell death by Fas/FasL or perforin pathways - CD4+ and CD8 + T cells recognize β-cells antigens indirectly by APCs and induces β-cells death mediated by surface receptors (Fas/FasL, TNF-α/TNF-R), cytokines produced by T cells, activation of macrophages and stimulation of their cytocidal activities, and stimulation of the production of cell death mediators. **T2D** - Extrinsic pathway: Fas-FasL leads to death-inducing DISC formation and activates caspase-8, leading to caspase-3 activation - Intrinsic pathway: Proapoptotic Bcl proteins are activated and are translocated to mitochondria to inactivate Bcl proteins and form pores in the mitochondrial membrane, facilitating the release of cytochrome c into the cytosol. Cytosolic cytochrome C interacts with apocaspase-9 and Apaf-1 to form an apoptosome, which in turn activates caspase-3.	**Inhibiting specific or broad-spectrum caspases**
**Necroptosis** Necroptotic cell death mediated by RIPK1/RIPK3 and MLKL may play a role in human diseases PMID: 31391333 PMID: 31783008	**Neurodegenerative diseases** AD, PD, ALS, Brain/spinal injury	**RIPK3-dependent injury** **RIPK1-dependent neuroinflammation** **PD**: RIPK1, RIPK3, and MLKL expression was upregulated in the substantia nigra of PD-derived postmortem tissue. **PD**: RIPK1, MLKL, and p-MLKL levels were increased in human AD brains. **MS**: RIPK1, RIPK3, and MLKL levels were increased in cortical lesions in human MS brain samples.	
	**Cardiovascular diseases** Heart disease, Stroke, Atherosclerosis, I/R Injury	**RIPK3-dependent injury** **Reduced ATP level** **Triggered** by Caspase8-dependent death receptor **Released DAMPs**: DNA and IL-6 **Core genes**: Positive: RIPK1/3 & MLKL; negative: cIAPs, ESCRT-III, PPM1B, LUBAC & AURKA **Aortic aneurysms**: RIPK1 and RIPK3 are increased in human abdominal aortic aneurysms. **Atherosclerosis**: The expressions of RIPK3 and MLKL are elevated in humans with unstable carotid atherosclerosis.	**Inducers** - zVAD-fmk, TNF-α & lipopolysaccharide **Inhibitors** Ac-YVAD-cmk, VX765, MCC950, isoliquiritigenin, glybenclamide & oridonin
	**Cancer** Breast cancer, Colorectal cancer, AML, Melanoma, Cell carcinoma, Leukemia, Glioblastoma, Lung cancer, Pancreatic cancer, Gastric cancer, Ovarian cancer, Cervical squamous cell, Carcinoma	**Modulation of oncogenesis** **Decreased/increased expression:** RIPK1 RIPK3 CYLD MLKL FADD	**Immune stimulation** - GSDMs **Epigenetic methods** - GSDMs **CCCR/CAR-T** - GSDME **Biorthogonal chemistry** - GSDMA
	**Renal disease** Acute kidney injury, Chronic kidney disease	**RIPK1-dependent injury** **RIPK3-dependent neuroinflammation**	
	**Pulmonary Disease** Acute lung injury, Chronic lung disease, Sepsis	**RIPK3-dependent injury** **RIPK1-dependent Neuroinflammation** **COPD**: pRIPK3, MLKL, and p-MLKL levels are increased in the lungs of patients with COPD. **IPF**: RIPK3 and p-MLKL levels are increased in the lungs of patients with IPF.	
	**Hepatic Disease** NAFLD/NASH, Acute hepatotoxicity, Hepatocarcinoma, Hepatitis	**RIPK1-dependent neuroinflammation** **Alcoholic hepatitis**: RIPK3 is increased in liver tissues of humans with alcoholic liver disease.	
	**Infectious diseases**	**Inhibition of the RIPK1 or RIPK3 activity** **Deletion of MLKL** **Deficiency of both RIPK3 and caspase‑8**	
	**Metabolic diseases** Obesity, NAFLD, Diabetes (T1D, T2D)	**Obesity and NAFLD:** - In NAFLD models, RIPK1, RIPK3, and MLKL are upregulated - In visceral adipose tissue of patients with obesity, levels of RIPK3 and p-MLKL are increased - RIPK3 expression has a positive correlation with p-MLKL and metabolic markers (blood insulin levels and HbA1C). **T1D:** - TNF-α binds to its receptor and forms the IIb complex - Excessive ROS production medicated by necrosome. **T2D:** - TNFR1 signaling - RIPK3 oligomerization MLKL phosphorylation.	**Inhibiting necroptotic components**: RIPK1, RIPK3, and MLKL
**Ferroptosis** Iron and ROS accumulation; inhibition of system xc − ; with decreased cystine uptake; GSH depletion and increased; NADPH oxidation release of arachidonic acid mediators PMID: 31248150 PMID: 33485988 PMID: 33495651	**Cardiovascular diseases** Heart disease Stroke Atherosclerosis, I/R Injury	**RIPK3-dependent injury** **Drop in ATP** **Triggered** by Caspase8-dependent death receptor **Released DAMPs**: DNA and IL-6 **Core genes**: Positive: RIPK1/3 & MLKL; negative: cIAPs, ESCRT-III, PPM1B, LUBAC &AURKA **Aortic aneurysms**: RIPK1 and RIPK3 are increased in human abdominal aortic aneurysms. **Atherosclerosis**: The expression of RIPK3 and MLKL is elevated in humans with unstable carotid atherosclerosis.	**Inducers** - zVAD-fmk, TNF-α & lipopolysaccharide **Inhibitors** Ac-YVAD-cmk, VX765, MCC950, isoliquiritigenin, glybenclamide & oridonin
	**Cancer** Breast cancer, Colorectal cancer AML, Melanoma, Cell carcinoma, Leukemia, Glioblastoma, Lung cancer, Pancreatic cancer, Gastric cancer, Ovarian cancer, Cervical squamous cell, Carcinoma	**Modulation of oncogenesis** **Decreasing/increasing expression of:** RIPK1 RIPK3 CYLD MLKL FADD	**Immune stimulation** - GSDMs **Epigenetic methods** - GSDMs **CCCR/CAR-T** - GSDME **Bioorthogonal chemistry** - GSDMA
	**Renal disease** Acute kidney injurym Chronic kidney disease	**RIPK1-dependent necroinflammation** **RIPK3-dependent injury**	-
	**Pulmonary Disease** Acute lung injury, Chronic lung disease, Sepsis	**RIPK3-dependent injury** **RIPK1-dependent neuroinflammation** **COPD**: pRIPK3, MLKL, and p-MLKL levels are increased in the lungs of patients with COPD. **IPF**: RIPK3 and p-MLKL are increased in the lungs of patients with IPF.	
	**Hepatic Disease** NAFLD/NASH. Acute hepatotoxicity. Hepatocarcinoma. Hepatitis	**RIPK1-dependent Neuroinflammation** **Alcoholic hepatitis**: The RIPK3 level is increased in liver tissues of humans with alcoholic liver disease.	
	**Infectious diseases**	**Inhibition of the RIP1 or RIP3** **Deletion of MLKL** **Deficiency of both RIP3 and caspase‑8**	
	**Circulatory system** I/R injury, Myocardial infarction, Heart failure	**Inhibition** of xCT & GPX4, GSH depletion, iron-dependent lipid peroxidation **Triggered** by iron overload, insufficient cellular reducing capacity **Activated inflammasome**: NLRP3, AIM2 & Pyrin **Core genes**: Positive: TFRC, ACSL4, GLS2, ALOX15, NCOA4, CARS, VDAC2/3, HSP90 & ALK4/5; negative: NRF2, GPX4, SLC7A11, HSPB1/5 & FANCD2 **Released DAMPs**: HMGB1	**Inducers** - Erastin, erastin analogs, sulfasalazine, sorafenib, glutamate, INF-γ, (1 S,3 R)-RSL3, statins, ML162, phospholipids with two PUFA tails **Inhibitors** - Liproxstatins, ferrostatins, Trolox, baicalein, vitamin E, α-tocopherol, zileuton, ciclopirox, deferiprone & dihydrobiopterin
	**Inflammation and Infection**	**GPX4** can inhibit the activation of arachidonic acid **NF-κB pathways** Reduce levels of ROS induced by lipid peroxidation	**Reduces GSH levels** - Upregulate GSH metabolic regulatory protein dipeptidase-1 (DPEP1)
	**Metabolic diseases** Diabetes (T1D, T2D)	**Diabetes and complications** - Multiple proteins involved in diabetes and complications: ACSL4, HMGB1, HIF-1α, HO-1, TRIM46, circ-PSEN1, NCOA4, and Nox2 Negative regulators: GPX4, NRF2, xCT, adenosine monophosphate-activated protein kinase, HSF1, NAF-1	**Inhibit ferroptosis pathway activation**
**Pyroptosis** Caspase-1 activation; Caspase-7 activation; secretion of IL-1β and IL-18	**Cancer** Melanoma, Breast cancer, Colorectal cancer, Gastric cancer, Hepatocellular carcinoma, Lung cancer, Cervical cancer, Leukemia PMID: 33776057	**Increased release of mature IL-1β and IL-18** **Roles of ROS/NF-κB/NLRP3/GSDMD signaling** **Lung cancer**: promoted by GSDMD **Liver cancer**: DFNA5 and NLRP3 **Breast and Gastric cancer**: GSDME and GSDMD attenuate diseases. **Colorectal cancer**: induced by GSDMC and GSDME; inhibited by GSDMD. **Melanoma cancer**: induced by GSDMC, HMGB1; inhibited by BRAF and MEK. **Acute myeloid leukemia**: GSDMD upregulates and represses leukemia progression	**Regulate:** - GSDMC-/B-/A-related pyroptosis - GSDME-based pyroptosis - GSDMD-mediated pyroptosis
	**Nervous system diseases** CNS infectious disease: Bacterial, viral, and parasitic infection, neurodegenerative disease: AD, PD, HD, and ALS; Stroke, Epilepsy, Neurodevelopmental disease PMID: 34838808	**Bacterial infection**: NLRP3, caspase-1/11-GSDMD signaling **Viral infection**: NLRP3 inflammasome, GSDMD **Parasitic infection**: alarmin IL-1α and GSDMD **Neurodegenerative disease**: ASC, NLRP3/4, GSDMD, caspase-1, -3, -7, -8, -11. **Stroke**: NLRP3, NLRC4, AIM2, GSDMD, caspase-1 **Epilepsy**: NLRP3, NLRP1, caspase-1 **Neurodevelopmental disease**: AIM2, GSDMD, NLRP3, ASC, caspase-1	**Targeting:** - NLRP3 inhibitors - ASC inhibitors - GSDMD inhibitors - GSDMD pore formation - GSDMD interaction with caspase GSDMD cleavage - Caspase inhibitors: Caspase-1/4 Caspase-1/4/5/11 Caspase-1
	**Infectious disease** PMID: 28462526	Caspase-1-dependent mechanism, Caspase-1-independent mechanism, cleavage of GSDMD to initiate pyroptosis, pro-inflammatory cytokines IL-1β and IL-18, alarmins and endogenous danger-associated molecular patterns, AIM2 inflammasome IFN signaling is a central modulator of pyroptosis induced by pathogens. **Bacterial infection** - Caspase-1 activation: Regulates phagosome maturation during gram-negative and gram-positive bacterial infection, promotes fusion between vacuoles containing bacteria and lysosomes, regulates pH of phagosomes to enhance+ killing of pathogens, and reduces cellular stiffness to control bacterial burden autonomously - Catalytic activity of caspase-1 and caspase-11 is essential for dampening the growth of bacteria. Caspase-11 regulates actin polymerization via cofilin to promote fusion between bacterial and lysosomes. **Viral, fungal, and protozoan infection** - Both DNA and RNA viruses activate the inflammasome and induce pyroptosis - Caspase-1 and caspase-11 against infection by influenza A virus and West Nile virus - HIV: DNA sensor IFI16 recognizes cytosolic viral DNA intermediates produced in human macrophages or CD4 T cells, which depletes CD4 T cells and promotes progress to AIDS in humans - Aspergillus fumigatus: Caspase-1 dependent response is crucial for the generation of protective cytokine IL-1β and IL-18 in a mouse model.	
	**Cardiovascular disease** Atherosclerosis, Ischemic heart disease, Diabetic cardiomyopathy, Cardiac hypertrophy, PMID: 30525268 PMID: 31337966	**Activation** of caspase-1 and GSDMD, GSDMD-N–induced pore formation, IL-1β release **Triggered** by activated inflammasomes (NLRP3, AIM2 & Pyrin): ROS **Released DMAPs**: HMGB1, ATP, IL-1β, and IL-18 **Core genes:** Positive: CASP1/11 & GSDMD; negative: PKA, ESCRT-III & GPX4	**Targeting NLRP3, caspase 1**
	**Autoimmune disease** Systemic lupus erythematosus, Rheumatoid arthritis, Inflammatory bowel disease, Sjogren’s syndrome, Dermatomyositis PMID: 35693810	**Switches to pyroptosis**: NLRP3, AIM2, and P2X7-NLRP3. **Roles** of canonical caspase-1-mediated pathway, noncanonical caspase-4/5-/11-mediated pathway, caspase-3-mediated pathway, and caspase-independent pathway	
	**Metabolic diseases** - Obesity, - NAFLD, - Diabetes (T1D, T2D)	**Obesity and NASH:** - Caspase-8 functions as upstream and downstream of the NLRP3 inflammasome - NLRP3 inflammasome-mediated activation of caspase-1. **Diabetes:** - NLRP3 inflammasome-mediated pyroptosis	**Targeting NLRP3 inflammasome pathway**

### Cancer

Mitochondrion involved in cell death has been implicated in cancer development. Physiologically, apoptosis is a protective defense mechanism that effectively inhibits tumor growth and eliminates neoplastic cells. A major mechanism triggering apoptosis involves elevated ROS levels in cancer cells resulting from heightened metabolic activity. As previously discussed, ROS at excessive levels disrupt mitochondrial functions, resulting in mitochondrial membrane depolarization and mPTP opening, which subsequently activates intrinsic apoptosis pathways. Moreover, membrane depolarization is a crucial event in the activation of TRAIL, a ligand that causes fragmentation of mitochondria in multiple human cancer cell lines, ultimately inducing apoptosis and necroptosis by activating caspases and RIPK1/RIPK3, respectively. Excessive levels of ROS can also promote necroptosis via RIPK1 oxidation and necrosome complex formation, followed by inflammatory and anticancer immune responses^85^. ROS generation also mediates ferroptosis. Given that they exhibit increased reliance on cellular iron levels, cancer cells are particularly susceptible to ferroptosis^86^. The ROS-induced ferroptotic cascade leads to oxidative stress within the cytoplasm, culminating in the release of cytokines and inflammatory mediators such as TNF-α and IL-6, which potentiate the immune system to combat cancer. Novel investigations have revealed that CD8^+^ T cells, the main forces of cancer eradication, can directly induce ferroptosis in cancer cells, highlighting the potential significance of ferroptosis in cancer treatment^87^. ROS may also trigger ferroptosis and promote NLRP3 inflammasome activation and subsequent pyroptosis. In addition to higher ROS levels, cancer cells may show greater susceptibility to apoptosis than normal cells. Genetic complexity, metabolic regulation, and stress responses drive cancers closer to apoptosis initiation. In response to oncogene activation, in multiple cancer types, such as chronic lymphocytic leukemia, melanoma, and lung cancer, the levels of proapoptotic proteins are increased, stimulating the oligomerization of Bax or Bak and culminating in MOMP and apoptosis^88^. Moreover, an upstream stimulator of apoptosis, p53, triggers the innate immune system pathway to drive both cell-intrinsic and cell-extrinsic tumor-suppressing activities^89^.

As a pivotal characteristic of cancer pathophysiology, tumor cell immune evasion is an intrinsic and vital process that facilitates cancer progression and metastasis due to inefficacy of host immune responses^90^. Among the intricate mechanisms involved in immune responses, mitochondria are core mediators that both inhibit and promote immune evasion^91^. Mitochondria inhibit immune responses by suppressing metabolism^92^ by regulating metabolic pathway activation^93^, affecting immune cell activity. This metabolic limitation, in turn, promotes tumor cell immune evasion, facilitating their unbridled proliferation. In addition, mitochondria also contribute to tumor cell immune evasion by releasing mtDNA and synthesizing antioxidants, which mitigate oxidative stress in cancer cells^94^. Intriguingly, recent research has revealed that nanotubes, which connect immune cells and cancer cells, enabling mitochondria to be hijacked from immune cells by cancer cells. This mitochondrial transfer promotes cancer cell metabolism while depleting immune cells, exhibiting a distinctive mechanism of immune evasion in cancer cellss^95^.

Mitochondrial cell death constitutes a robust barrier that complements MQC in defense against cancers. This outcome is attributable not only to the direct clearance of cancer cells but also to PCD-induced immune attack. The induction of tumor-associated antigen release and promotion of immune-related factor expression induces immunogenic cell death in cancers. In response, however, a myriad of mechanisms in cancer cells mitigates these defense systems; in the crucial response, cancer cells escape PCD as well PCD-induced immune responses. This immune evasion is a pivotal characteristic of cancer pathophysiology. Notably, the dysregulation of apoptosis is a hallmark of numerous malignancies, such as follicular lymphoma and osteosarcoma, as indicated by the upregulation of anti-apoptotic Bcl-2 or Bcl-xL protein expression and the loss of pro-apoptotic BH3 protein expression. Similarly, the expression of proapoptotic effectors, including Bax and Bak, is commonly downregulated in colorectal, lung, and breast cancers^96^. In other cases, insufficient release of cyt *c* or an imbalanced redox state induced hinders caspase activation^97^. Moreover, cells in lung and colon cancers release mtDNA into the extracellular milieu, and this mtDNA is then phagocytosed by nearby immune cells. After internalization, mtDNA triggers immune cell apoptosis, facilitating immune evasion^94^. Furthermore, a cytotoxic T lymphocyte (CTL) mechanism called “additive cytotoxicity” and is induced via sublethal damage, and it involves perforin-dependent membrane pore formation and nuclear envelope rupture, driving cancer cells to undergo apoptosis. Notably, one of the primary mechanisms that thwarts an immune attack involves the upregulation of immune checkpoint molecules such as PD-L1, which significantly inhibits the activity of CTLs and hence indirectly suppresses apoptosis. In addition to apoptosis, cancer cells leverage multifarious mitochondrion-associated nonapoptotic PCD modalities to realize immune evasion. Factors that inhibit the immune response are secreted from cancer cells and facilitate interference with immune recognition and thus promote necroptosis. The absence or epigenetic silencing of RIPK3 expression via hypermethylation has been documented to inhibit necroptosis in various cancers, such as lung, gastric, ovarian, and colorectal cancer^98^. In addition, decreased intracellular free ferrous ions and lipid peroxidation rates in cancer cells inhibit immunogenic ferroptosis and prevent inflammatory immune responses. Mutations in BAP1, a nuclear deubiquitinating enzyme that is highly active in various human cancers, also facilitates tumor cell escape from ferroptosis^99^. Similarly, cancer cells modulate the NF-κB signaling cascade via diverse mechanisms, suppressing innate immunity-triggered pyroptosis. Although low GSDME expression is commonly detected in several cancers, including lung and gastric cancers and is acknowledged to be a pivotal therapeutic target for counteracting pyroptosis evasion and advancing cancer management, the mechanisms underlying its reduced levels are unknown^100^.

Notably, certain type of mitochondrial cell death might, to some degree, contribute to the proliferation and metastasis of cancer cells. For example, lymphoma and glioblastoma cells exhibit remarkable rates of spontaneous apoptosis, which are correlated with poor prognosis^101^. In contrast, the facilitated release of a minimal level of cyt *c* has been observed to induce low yet continuous caspase-9 activation, thereby promoting genomic instability in viable cells and facilitating oncogenic transformation^102^. MOMP-dependent global mRNA decay may lead to preferential transcription of Bcl-2 and Bcl-xL mRNA, particularly under mild or transient stress conditions, to support cancer progression^103^. Additionally, necroptosis may fuel carcinogenesis by inducing adaptable immunosuppression, as indicated by the upregulation of RIPK1, RIPK3, and MLKL in some aggressive cancers, including lung cancer and glioblastoma.

Collectively, these findings underscore the intricately involved and elusive nature of mitochondrial cell death in cancer eradication and tumor cell immune evasion, necessitating ongoing efforts to characterize its complex role. Moreover the need to tailor cancer treatments targeting mitochondrion-associated PCD based on specific phenotypes in certain circumstances is increasing.

### Neurodegenerative diseases

Various neurodegenerative diseases have been linked to different mechanisms underlying mitochondrion-related dysfunction, such as apoptosis, Ca^2+^ overload, and ROS generation^104^, since neurons largely rely on mitochondria for energy production and Ca^2+^ buffering and, therefore, are very vulnerable to mitochondrial abnormalities.

Alzheimer’s disease (AD) is a canonical paradigm that supports the claim that mitochondrial dysfunction is associated with pathology. Amyloid β (Aβ) accumulation, a key initiator of AD, induced mitochondrial Ca^2+^ overload in conjunction with increasing hyperphosphorylated tau protein levels, triggering sustained mPTP opening and disrupting mitochondrial homeostasis to stimulate the caspase cascade in apoptosis^105^. In line with these findings, apoptosis has also been associated with acute brain injury. Most neurons in an ischemic region undergo apoptosis characterized by typical apoptotic features, including apoptotic signaling pathway activation and typical morphological alterations. Additionally, MOMP and apoptosis are likely involved in the development of chronic neurodegenerative diseases^106^. Shifts in the ER stress response or Ca^2+^ buffering to activate mitochondrial proteases and cause mitochondrial membrane permeability have been widely described in cells and transgenic AD mouse models.

An increasing body of evidence suggests that nonapoptotic PCD may play an important role in the pathophysiology of neurological diseases such as AD and delayed ischemic injury. Notably, RIPK motifs promote the assembly and accumulation of Aβs, which in turn form active signaling complexes that mediate necroptosis^107^. Multiple studies have established the role of ferroptosis in neurotoxicity and brain injury; in fact, ferroptosis was initially called glutamate-induced neuronal excitotoxic death, which was blocked by a ferroptosis inhibitor^96^. Given that high levels of extracellular glutamate trigger ferroptosis and that glutamate neurotoxicity contributes to stroke and other neurodegenerative illnesses, ferroptosis likely contributes to the pathophysiology of multiple brain disorders. Genetic studies have supported the idea that the effects of conditional Gpx4 deletion resemble the effects of neurodegeneration, although the mechanism is unclear^108^.

### Cardiovascular diseases

Similar to neurodegeneration, mitochondrion-associated PCD is a key regulator of different CVDs. Although apoptosis is not the sole mode of cell death in CVDs, it is believed to contribute to various disorders, such as heart failure, atherosclerosis, and aneurysm^109^. Apoptotic cardiomyocyte death tends to be complicated by irrevocable congestive heart failure, consistent with findings showing that inhibiting apoptosis can attenuate contractile dysfunction and ventricular dilation, both of which are hallmarks of heart failure^110^. Endothelial cells that undergo apoptosis but are not eliminated in a timely manner, contribute to atherosclerosis^111^. Plaque formation may be facilitated by an increased propensity of apoptotic cells to coagulate and adhere to platelets; as a result, apoptosis may contribute to plaque instability and rupture. Moreover, apoptosis has been thought to accelerate aneurysm development by depleting medial vascular smooth muscle cells, an outcome that caspase inhibitors^100^ may prevent.

In addition to apoptotic PCD, necroptosis induced by ischemia/reperfusion (I/R) or oxidative stress contributes to heart failure, predominantly through ATP depletion and mPTP opening, as indicated by attenuated injury in CypD-deficient mice and increased levels of RIPK3 in ischemic tissues post-I/R. Notably, pyroptosis has been more comprehensively investigated in CVD than in any other organ system. As a core regulator, NLRP3 is stimulated by oxidized low-density lipoprotein, which induces the inflammation evident in pyroptosis. Similarly, lipids accumulate following arterial endothelial injury, and large quantities of caspase-1 are seen in plaques deposited after the development of atherosclerosis. In diabetic heart disease, high glucose levels can trigger ROS overexpression and accelerate NLRP3 inflammasome formation, indirectly inducing pyroptosis. Recent research has also suggests that some nonapoptotic PCD modalities may be activated during ischemia. NLRP3 inflammasome inhibitors strengthen cardiac tolerance to ischemic insult, providing convincing justification for further targeting ferroptosis in therapeutic trials^112^. Additionally, inhibiting ferroptosis attenuates acute and chronic ischemia and protects against heart failure induced by iron overload^113^.

### Metabolic diseases

Metabolic diseases are among the most common disorders related to mitochondrial dysfunction. Mitochondrion-associated cell death has been cited as a critical factor in regulating the survival of different cells under pathological conditions.

In persons with obesity and NASH, adipocyte and hepatocyte cell death via apoptosis plays a central role pathogenesis^114,115^. In the adipose tissues of patients with obesity, the expression of caspase-3, caspase-7, caspase-8, and caspase-9 is increased, and that of Bcl-2is decreased, correlating with insulin-resistance progression^116^. Excessive accumulation of lipid species results in adipocyte death, triggering an inflammatory macrophage response. The elevation of circulating FFAs also contributes to an accelerated lipid metabolism rate in the liver and subsequent development of NAFLD. During this disease progression, adipose tissue secretes inflammatory cytokines and exacerbates liver inflammation and hepatocyte death^117^. In this context, the mechanism underlying apoptosis is activated via caspase-dependent extrinsic and intrinsic pathways. Both pathways initiate MOMP and caspase-2 activation, resulting in caspase-3 and caspase-7 activation and apoptosis. In addition to those in the apoptosis pathway, factors in other cell death pathways, such as +pyroptosis and necroptosis pathways, engage in crosstalk with caspases to regulate cell death mechanisms in metabolic diseases^118^. Pyroptosis is initiated when gasdermin D is cleaved by caspase-1 or caspase-4, caspase-5, and caspase-11. Caspase-8 regulates the activation of the NLRP3 inflammasome, a critical component in pyroptotic cell death. The activation of the NLRP3 inflammasome is also associated with necroptosis induction. Because of this crosstalk, interventions to block one pathway may affect other pathways to mediate disease pathogenesis.

Diabetes mellitus is a common metabolic disorder in which pancreatic β-cell destruction is the main cause of β-cell failure and the onset of diabetes. In response to different stimuli, cells undergo different types of cell death, which modulates the survival of β-cells during disease progression. Apoptosis is the predominant mechanism associated with β-cell loss in Type 1 diabetes and a reduced β-cell population in Type 2 diabetes^119–122^. In Type 1 diabetes, β-cell failure is mediated by an immune response characterized by mononuclear and T cells interacting with antigen-presenting cells to generate a high level of inflammatory cytokines, chemokines, and other triggering factors, leading to apoptotic cell death. Three main mechanisms have been reported to be important in diabetes: 1) the expression of Fas on CD8^+^ T cells and FasL on β-cells, 2) the secretion of proinflammation factors (IL-1β, TNF-α, and IFN-γ) by the immune cells that have infiltrated in islets, and 3) the production of ROS by macrophages^123^. In Type 2 diabetes, multiple causes, such as glucotoxicity, lipotoxicity, and islet amyloid polypeptide aggregation, contribute to β-cell dysfunction and β-cell death. ER stress pathways (the PERK, IRE1, and ATF6 pathways) are activated in response to these factors. When ER stress is prolonged, increases in the level of the proapoptotic factor CHOP leads to ROS production, mitochondrial dysfunction, and apoptosis^124^. Apoptotic β-cell death results from an imbalance between proapoptotic Bcl-2 protein (Bad, Bid, Bik, and Bax) and antiapoptotic Bcl family protein (Bcl-2 and Bcl-xL) levels. In addition to apoptosis, other cell death modalities are involved in the pathogenesis of Type 1 and Type 2 diabetes. In Type 1 diabetes, β-cells are susceptible to necrosis through the activation of cytokine signaling. After TNF-α binds to its receptor and forms a necrosome complex, ROS production and DNA fragmentation are initiated, contributing to necroptosis^125^. Several studies have reported that NLRP3-mediated pyroptosis promotes the pathogenesis and progression of Type 1 diabetes^126^. Regarding Type 2 diabetes, necroptosis, ferroptosis, and pyroptosis contribute to β-cell death regulation. In particular, ferroptosis may underlie β-cell loss related to Type 2 diabetes, as indicated by an increased risk for metabolic syndrome in individuals with elevated serum ferritin levels. A report showed that necroptosis was the previously unknown mechanism underlying β-cell death in response to islet amyloid formation. NLRP3 inflammasome-mediated pyroptosis also appears to contribute significantly to the development of Type 2 diabetes and insulin resistance^122^. The activation of the NLRP3 inflammasome can be induced by various factors, such as high glucose, fatty acids, ROS, and IAPP levels^127,128^.

Mitochondrion-associated cell death is complicated and plays an important pathophysiological role in regulating metabolic diseases. Understanding the relationships between different pathways and the underlying control mechanisms is critical to prevent cell death in the pathogenesis of these diseases.

### Other diseases

Almost all PCD modalities have been implicated in the pathogenic progression of digestive diseases to some extent, with some forms critical to the severity and prognosis of diseases. A key mediator of the extrinsic apoptosis pathway mediator for typical apoptotic cascades as well as initiator of signaling cascade reactions of necroptosis, TNF-α is extensively involved in the development of multiple diseases, such as inflammatory bowel disease (IBD), spontaneous colitis^129^, alcoholic liver disease, and NAFL/NASH^130^; notably, NAFL/NASH has been proven to be triggered by CyPD-induced mPTP opening, consistent with caspase-3-initiated caspase-6 cleavage and consequent cyt c release. In addition, multiple pathways are clearly involved in necroptosis and ferroptosis, albeit the molecular level details are unclear. Interestingly, the protective effect conferred by caspase-11-dependent pyroptosis on IBD and colitis was proposed to be independent of IL-1β or IL-18 action, indicating unique underlying pathways that need to be further explored.

PCD is intricately linked to inflammation as both a regulator and an endpoint. A pattern recognition receptor member, absent in melanoma 2 (AIM2), has ben reported to activate caspase-3, coupling with NLRP3 to induce caspase signaling in apoptosis. This process is mediated by caspase-1, which drives proinflammatory secretion to induce pyroptosis. The dysregulation of proinflammatory secretion may lead to hereditary autoinflammatory disorders, as evidenced IL-1β prompting chemokine overexpression and leukocyte migration. In addition, IL-18 has been shown to enhance the required for the activation of macrophages and T cells. Additionally, necroptosis is a key initiating event of inflammation, with secreted DAMPs the primary sources by which RIPK3 drives the inflammatory response following MLKL insertion into the plasma membrane. RIPK3 also drives inflammasome assembly to activate caspase-1 and caspase-11. As an RIPK3 regulator, RIPK1 increases circulating the IL-1α level to induce TNF production and the induction of spontaneous inflammatory symptoms via autocrine signaling.

Recent research has highlighted the typical phenotype acquired by cells for which PCD is a defense mechanism against infection. After viral infection of cells, apoptosis can be initiated by granzymes released from activated cytotoxic T lymphocytes and natural killer cells. Necroptotic RIPK3 is activated to limit viral replication. Remarkably, apoptosis and necroptosis seem to be linked to an immune signaling network to confer protection onto cells. Caspase 8 is an innate immune sensor ensconced within the TNF receptor and toll-like receptors in response to viral insults. At the same time, it interacts with RIPK1-RIPK3 to form the triangular guard system to protect each other and eliminate infections. As an auxiliary mechanism, pyroptosis increases viral susceptibility via AIM2 and removes pathogens via caspase-1 activation^131^.

### Clinical trials

As outlined above, mitochondrion-associated PCD is generally associated with the course of multiorgan disorders. In this circumstance, targeted regulatory biomarker or signaling pathway candidates have piqued the interest of scholars, and an increasingly number have been entered into early clinical trials (Table 2). For instance, deferiprone is predicted to attenuate the increase in iron levels in the substantia nigra via ferroptosis inhibition, providing promising therapeutic benefits for several neurodegenerative disorders, such as AD and Parkinson’s disease. Navitoclax, a dual inhibitor of Bcl-2 and Bcl-xL, has been entered into a trial exploring convincing cancer therapies. Similar trials are being planned to evaluate newly developed PCD mediators, with hundreds of registered trials recruiting patients or ongoing. Therefore, it is anticipated that these clinical trials based on extensive PCD research will lead to considerable breakthroughs for disease treatment.

**Table. 2:** Current list of clinical trials targeting mitochondria-associated programmed cell death in human diseases.

Mitochondrion-associated Cell death	Diseases	Drugs/mode of action	Status of clinical trials	Developers	References and/or clinical trial registration number
**1. Neurodegenerative diseases**					
**Apoptosis**	GBM	**Carboplatin**/DNA-damaging agent triggering apoptosis. Evaluated in combination with Bevacizumab (VEGF inhibitor).	Phase II (122), completed	Roche Products Australia Pty Ltd.	ACTRN12610000915055 PMID: 26130744
		**Olaparib**/PARP inhibitor sensitizes GBM cells to death receptor-mediated apoptosis induced by TRAIL. These agents all induce apoptosis in malignant cells.	Phase I/IIa (79), recruiting	Centre Francois Baclesse	NCT03212742 PMID: 30832617
**Necroptosis**	ALS	**DNL747**/RIPK1 inhibitor	Phase I (15), terminated	Sanofi	NCT03757351
	AD		Phase I (16), completed	Denali Therapeutics Inc.	NCT03757325
**Ferroptosis**	PD	**Deferiprone**/iron chelator	Phase II (40), completed	University Hospital, Lille	NCT00943748 PMID: 24251381
			Phase II (22), completed	Imperial College London	NCT01539837 PMID: 28469157
	AD	**Deferiprone**/iron chelator	Phase II (171) ongoing	Neuroscience Trials Australia	NCT03234686
	ALS	**Deferiprone**/iron chelator	Phase II (23), completed	University Hospital, Lille	NCT02164253
			Phase II (240) ongoing		NCT03293069
	PD	**CuII(atsm)**/radical scavenger	Phase I (31), completed	Collaborative Medicinal Development Pty Limited	NCT03204929
	ALS		Phase I (50), completed		NCT02870634
			Phase I (28) Ongoing		NCT03136809
			Phase II (80) ongoing		NCT04082832
			Phase II (70) ongoing		NCT04313166
**2. Cancer**					
**Apoptosis**	Ovarian cancer; endometrial cancer	**Selinexor**/induces apoptosis by decreasing NF-κB activity	Phase I (23), completed	Memorial Sloan Kettering Cancer Center	NCT02269293 PMID: 28314490
	Breast cancer	**Pentoxifylline**/induces apoptosis by decreasing NF-κB	Phase 2 (48), terminated	University Health Network, Toronto	NCT00188669
		**Bortezomib**/induces apoptosis by decreasing NF-κB	Phase 2, completed	Northwestern University	NCT00028639 PMID: 33775688
	CLL, melanoma, solid tumors	**Navitoclax**/dual Bcl-2 and Bcl-xL inhibitor	Phase I/II (130), recruiting	National Cancer Institute (NCI)	NCT02079740
			Phase I (44), active		NCT02143401
			Phase I/II (75), active		NCT01989585
			Phase I (50), active		NCT02520778
	SCLC, solid tumors	**APG-1252**/dual Bcl-2 and Bcl-xL inhibitor	Phase I (24), terminated	Ascentage Pharma Group Inc.	NCT03387332
	NHL, multiple myeloma	**S55746 (BCL201)**/selective Bcl-2 inhibitors	Phase I (20), completed	Novartis Pharmaceuticals	NCT02603445
			Phase I (65), completed	Institut de Recherches Internationales Servier	NCT02920697
	Solid tumors	**ABBV-155**/Bcl-xL inhibitors	Phase I (176), recruiting	AbbVie	NCT03595059
	NHL, AML	**APG-2575**/Selective Bcl-2 inhibitors	Early phase I (90), recruiting	Ascentage Pharma Group Inc.	NCT03537482
			Phase I (74), recruiting		NCT03913949
		**AMG 176**/MCL-1 inhibitors	Phase I (175), recruiting	Amgen	NCT02675452
		**AMG 176**/MCL-1 inhibitors **MIK665 (S64315)**/MCL-1 inhibitors	Phase I (9), terminated (Safety)	AbbVie	NCT03797261
			Phase I (31), completed	Novartis Pharmaceuticals	NCT02992483
		**MIK665 (S64315)**/MCL-1 inhibitors **AZD5991**/MCL-1 inhibitors	Phase I (40), recruiting	Institut de Recherches Internationales Servier	NCT03672695
			Phase I (38), completed	Institut de Recherches Internationales Servier AstraZeneca	NCT02979366
			Phase I (70), terminated		NCT03218683
		**LCL161**/IAP inhibitors and SMAC mimetic antagonists	Phase I (34), terminated	SCRI Development Innovations, LLC	NCT02649673
	Colorectal cancer, multiple myeloma, polycythemia vera, myelofibrosis	**LCL161**/IAP inhibitors and SMAC mimetic antagonists **Birinapant (TL32711)**/IAP inhibitors and SMAC mimetic antagonists	Phase II (53), completed	Anderson Cancer Center	NCT02098161
			Phase I (26), completed	Novartis Pharmaceuticals	NCT03111992
			Phase I (34), recruiting	NCI	NCT03803774
	Advanced solid tumors, NHL	**Birinapant (TL32711)**/IAP inhibitors and SMAC mimetic antagonists **Sorafenib**/GPX4 inactivation due to GSH depletion (Class I FINs) – targeting system XC-	Phase I/II (34), terminated	Medivir	NCT02587962
			Phase II (86), completed	Chinese Academy of Medical Sciences	NCT03535259 PMID: 34977874
**Ferroptosis**	HCC, RCC, NSCLC, PDAC	**Sorafenib**/GPX4 inactivation due to GSH depletion (Class I FINs) – targeting system XC- **Statins**/Reduce selenoproteins (such as GPX4) and CoQ10 biosynthesis	Phase II (500), recruiting	Wake Forest University Health Sciences	NCT02559778
	Neuroblastoma		Phase II (342), completed	Eastern Cooperative Oncology Group	NCT00064350
	Non-small cell lung cancer		Phase I/II (74), recruiting	Anderson Cancer Center	NCT03247088
	Acute myeloid leukemia		Phase III (314)	Peking Union Medical College Hospital	NCT03971019 PMID: 23471651
	Breast cancer	**Continue PD-1**/PD-L1 Inhibitors treatment	Phase III (578), recruiting	Antoinette J Wozniak	NCT04157985
	Fibrosarcoma, Bladder cancer	**APR-246 (eprenetapopt)**/GSH depletion, thioredoxin inhibition	Phase I (37), completed	Aprea Therapeutics	NCT04383938
	AML, non-small cell lung cancer, bladder cancer	**Altretamine**/GPX4 inhibition	Phase I/II	Roswell Park Cancer Institute	NCT00002936
	Lymphoma, sarcoma	**Bromelain**/upregulation of ACSL4	Phase II (100), Unknown	Oeyama-Moto Cancer Research Foundation	NCT02340845
	Colorectal cancer	**Artesunate**/GPX4 inactivation due to GSH depletion (Class I FINs) – targeting glutathione S-transferase	Phase I (19), completed	Georgetown University	NCT02353026 PMID: 29392450
	Solid tumors	**Artesunate**/GPX4 inactivation due to GSH depletion (Class I FINs) – targeting glutathione S-transferase **Eprenetapopt**/targeting p53	Phase I (23), completed	Heidelberg University	NCT00764036
	Metastatic breast cancer		Phase II (200), recruiting	The 108 Military Central Hospital	NCT03093129 PMID: 26137537
	Colorectal cancer (Stage II/III)		Phase II (78), recruiting	Frantz Viral Therapeutics, LLC	NCT04098744
	Cervical cancer		Phase II (200), recruiting	University of London	NCT02633098
	Colorectal cancer (Stage II/III)		Phase I/II (100), suspended	Aprea Therapeutics	NCT04419389
	Non-Hodgkin lymphomas	**Eprenetapopt**/Targeting p53 **Sulfasalazine**/increase in ROS levels and a depletion of GPX4 and system xc‑ levels	Phase III (154), completed	Aprea Therapeutics Haukeland University Hospital	NCT03745716
	Myelodysplastic syndromes		Phase I/II (247), completed		NCT02098343 PMID: 22965953
	High-grade ovarian cancer		Phase I/II (37), completed		NCT04383938
	Solid tumors		Phase I (51), completed		NCT04214860
	P53-mutant myeloid malignancies		Phase I (24), recruiting		NCT04205357
	Glioblastoma	**Sulfasalazine**/increase in ROS levels and a depletion of GPX4 and system xc‑ levels **Buthionine sulfoximine**/GPX4 inactivation due to GSH depletion (Class I FINs)	Phase I (9), completed	University of Alabama at Birmingham	NCT01577966
	Glioma		Phase I (18), completed	Children’s Hospital Los Angeles	NCT00002730
	Neuroblastoma	**Fluvastatin**/GPX4 inactivation/depletion (Class II, III FINs) – targeting HMG-CoA reductase	Phase II (35), completed	University of California, San Francisco	NCT00416403
	Breast cancer	**Withaferin A**/GPX4 inactivation/depletion (Class II, III FINs) – targeting GPX4	Phase II (24), unknown	Tata Memorial Hospital	NCT00689195
	Osteosarcoma	**Actinomycin-D, doxorubicin, topotecan and bleomycin**/Caspase1/Caspase-3/GSDMD/GSDME/DFNA/eEF-2K	Not applicable, active, not recruiting	Children’s Hospitals and Clinics of Minnesota	NCT01464606
**Pyroptosis**	Pleuropulmonary blastoma	**Metformin**/miR-497/PELP1/Caspase-1/GSDMD	Phase II (93), completed	NCI	NCT01447927
	Esophageal Cancer	**Docosahexaenoic acid**/Caspase-1/GSDMD/IL-1β/HMGB1	Phase II (65), completed	NCI Sixth Affiliated Hospital, Sun Yat-sen University	NCT01849250
	Breast cancer	**5-fluorouracil**/Caspase-3/GSDME	Phase II (136), recruiting		NCT04358341
	Gastric cancer	**Magnesium glycinate**/calcium: magnesium balance, microbiota, and necroptosis and inflammation	Not applicable (240), active	Vanderbilt University Medical Center	NCT04229992 NCT01105169
**Necroptosis**	Colorectal cancer	**Aspirin**/MLKL	Phase II (81), active	NCI	NCT02965703
	Colorectal adenoma	**Birinapant**/MLKL	Phase I (34), recruiting	NCI	NCT03803774
	Head and neck squamous cell carcinoma	**Pembrolizumab (GSK3145095)**/RIPK1	Phase II (8), terminated	GlaxoSmithKline	NCT03681951
	Neoplasms, pancreatic	**Olaparib**/inhibits parthanatos by decreasing PARP	Phase I (98), active Phase I (197)	AstraZeneca	NCT00516373 NCT00777582 PMID: 27169564
**Parthanatos**	Breast cancer	**Niraparib**/inhibits parthanatos by decreasing PARP	Phase I (21), completed Phase I (113), completed	Tesaro, Inc.	NCT03329937 NCT00749502 PMID: 23810788
		**Rucaparib**/inhibits parthanatos by decreasing PARP	Phase II (78), completed Phase II (41), completed	Cancer Research UK UNICANCER	NCT00664781 NCT02505048
	Breast cancer, ovarian cancer	**Veliparib**/inhibits parthanatos by decreasing PARP	Phase I (16), completed Phase I (98), completed	AbbVie NCI	NCT02210663 NCT00892736 PMID: 28665051
	Breast cancer	**Talazoparib**/inhibits parthanatos by decreasing PARP	Phase I (36), completed Phase I (113), completed	NCI Pfizer	NCT01989546 NCT01286987
**3. Infectious diseases**					
**Apoptosis**	Hepatitis C, chronic	**VX-799**/small-molecule caspase inhibitor	Phase I, unknown	Vertex/Serono	Unknown
	Septic organ failure	Predicted mortality of necroptosis in a septic patient	Not applicable (72), completed	Universitas Sriwijaya	NCT04169412
**Necroptosis**	Sepsis	**SAR443122**/RIPK1	Phase I (68), completed	Sanofi	NCT04469621
	Corona virus infection	Standard treatments for sepsis	Not applicable (100), recruiting	Nanjing First Hospital, Nanjing Medical University	NCT05410665
**Pyroptosis**	Sepsis	Pyroptosis rate	Not applicable (100), recruiting	Jianfeng Xie	NCT04427371
**4. Cardiovascular diseases**					
**Apoptosis**	Heart disease	**Minocycline**/alters the mitochondrial membrane potential with apoptotic factor protein expression (JNK/p-JNK and caspase-3)	Phase I/II (60), completed	David Hess	NCT00630396
	Acute stroke	**Minocycline**/alters the mitochondrial membrane potential with apoptotic factor protein expression (JNK/p-JNK and caspase-3) MCI-186/	Phase II/III (100), recruiting	Stony Brook University	NCT03320018
	Ischemic stroke		Phase II (36), completed	Mitsubishi Tanabe Pharma Corporation	NCT00821821
	Acute ischemic stroke	**Erythropoietin**/activates the PI3K/Akt pathway	Phase III (138), completed	Deutsches Herzzentrum Muenchen	NCT00390832
	Myocardial infarction	**Levosimendan**/soluble apoptosis signaling molecules Fas/Fas Ligand; reduction in major pro-inflammatory cytokine (TNF-α, IL-6) levels	Phase III (246), unknown	Dr. Gerhard Pölzl	NCT03437226
	Heart failure	**Puerarin**/induces the production of Reduces the production of ROS and NOX4	Phase II (217), completed	The University of Hong Kong	NCT03676296 PMID: 29427658
**Ferroptosis**	Heart failure	**Dexrazoxane**/inhibits iron overload	Phase I (12) terminated	Medical City Children’s Hospital	NCT02519335
	Heart defects, congenital	**Deferoxamine**/inhibits iron overload	Phase II (60), completed	Novartis Pharmaceuticals	NCT01254227
	Cardiac iron overload	**Deferiprone**/inhibits iron overload	Phase IV, completed	Ospedale Microcitemico	NCT00800761
	Iron overload cardiomyopathy	**Puerarin**/unknown	Phase II (217), completed	The University of Hong Kong	NCT03676296
	Heart failure	**Sulfasalazine** system Xc-	Not applicable (60), completed	Boston University	NCT00554203
	Coronary artery disease	**Nicotine**/ROS‐NLRP3‐ASC	Phase II (152), completed	National Institute on Drug Abuse (NIDA)	NCT00469885
**Pyroptosis**	Cardiovascular disease	**Nicotine**/ROS‐NLRP3‐ASC	Phase II (152), completed	NIDA	NCT00469885
**5. Digestive diseases**					
**Apoptosis**	Crohn’s Disease	Pathophysiology of NEC by using human-entered biorepository	Not applicable (18), unknown	Fondazione IRCCS Ca’ Granda, Ospedale Maggiore Policlinico	NCT04549727
**Necroptosis**	Enterocolitis, necrotizing	**GSK2982772**/RIPK1	Phase II (36), completed	GlaxoSmithKline	NCT02903966
	Colitis, ulcerative	**GSK2982772**/RIPK1	Phase II (36), completed	GlaxoSmithKline	NCT02903966
**6. Autoimmune diseases**					
**Apoptosis**	Rheumatoid arthritis	**Infliximab (Remicade)**/anti-inflammatory, also induces apoptosis in macrophages	Not applicable (1061), completed	Janssen Korea, Ltd., Korea	NCT00760669
	Arthritis, rheumatoid	**GSK2982772**/RIPK1	Phase II (65), completed	GlaxoSmithKline	NCT02776033
**Necroptosis**	Psoriasis	**GSK2982772**/RIPK1 **SAR443820**/RIPK1	Phase II (52), completed	GlaxoSmithKline Sanofi	NCT02858492
	Arthritis, rheumatoid		Phase I (45), completed		NCT03266172
	MR. IR, PK, and PD		Phase I (14), completed		NCT04982991
	Multiple sclerosis, healthy subjects	**SAR443122**/RIPK1	Phase II (88), recruiting	Sanofi	NCT04781816
	Cutaneous lupus erythematosus				
**7. Metabolic disease**					
**Apoptosis**	Obesity and diabetes	**Pralnacasan VX-740**/Caspase-1 inhibitor	Preclinical *ob/ob* mouse	Vertex Pharmaceuticals	PMC3174591 PMC3683568
	Obesity and NASH	**Ac-YVAD-cmk**/Caspase-1 inhibitor	Preclinical obese and NASH mouse model	NonPharmaceuticals	PMC5022108
	NASH	Caspase-1, -8, -9 inhibitor	Phase II NASH/completed	Gilead Sciences	PMC3779694
	Obesity, NAFLD, NASH	**Emricasan IDN-6556; PF-03491390**/broad-spectrum pancaspase inhibitor	Preclinical obese and NASH mouse model Phase II in NAFLD/completed Phase II in NASH fibrosis/active, not recruiting Phase II in decompensated NASH cirrhosis/active, recruiting Phase II NASH cirrhosis and severe portal hypertension	Conatus Pharmaceuticals	PMID24750664 NCT02077374 NCT02686762 NCT03205345 NCT02960204
	NASH and NAFLD	**VX-166**/broad-spectrum pancaspase inhibitor	Preclinical mouse models of NASH and NAFLD	Vertex pharmaceuticals	PMID20557969 PMID19676126
	Diabetes	**EP1013**/broad-spectrum pancaspase inhibitor	Preclinical islet-transplanted mice	EpiCept Corporation	PMID18356409 PMID20332344
**Necroptosis**	Prediabetes	**Necrostatin-1**/RIPK1 inhibitor	Preclinical prediabetes rats		PMID34265741
	NAFLD	**RIPA-56**/RIPK1 inhibitor or necrosulfonamide/MLKL inhibitor	In vivo high-fat diet-fed mice		PMID31760070
**Ferroptosis**	Diabetes and its complications	**Quercetin, curcumin, cryptochlorogenic acid, epigallocatechin-3-gallate, melatonin, ferrostatin-1, deferoxamine, hepcidin, liproxstation-1, rosiglitazone, baicalein, sterubin**/ferroptosis inhibitors	In vitro pancreatic β-cells and in vivo diabetic rat and mouse models		PMID33324924 PMID34487731 PMID20586147 PMID28216051 PMID26700463 PMID17569207 PMID26216672 PMID24614112 PMID30615092 PMID31102787 PMID23483669 PMID31574461
**Pyroptosis**	NAFLD, NASH	**MCC950**/NLRP3 inflammasome inhibitor	Preclinical obese diabetic mice		PMID28167322
	Obesity, NAFLD, NASH	**CY-09**/NLRP3 inflammasome inhibitor	In vivo high-fat diet-fed mice		PMID29021150 PMID33213837
	NAFLD	**Glibenclamide**/NLRP3 inflammasome inhibitor	In vivo streptozotocin-induced nonalcoholic fatty liver disease in rats		PMID31834465
	NAFLD	**Parthenolide**/NLRP3, AIM2, NLRC4, MLRP1 inhibitor	In vivo NAFLD rat model		PMID28555525

## Perspectives

In this review, we have articulated the crucial roles of mitochondria in maintaining homeostasis via quality control networks and regulation of cell fate mediated via mitochondrion-associated PCD. In addition to summarizing the established pathways involved in cell disassembly mediated by apoptotic signaling, we highlighted the involvement of mitochondria in several nonapoptotic PCD modalities, and similar to apoptosis, they associated with considerable disease progression. Notably, it is increasingly evident that cell death modality pathways engage in crosstalk. These interactions involve mitochondria, and although their interplay has been described in broad strokes, the molecular details still need to be identified. In the future, in-depth studies should be focused on identifying novel forms of mitochondrion-associated PCD and characterize the involvement of these death modalities in disease pathogenesis. In addition, PCD-based therapy must be developed and evaluated in future clinical trials.
